# Improved secretory expression of lignocellulolytic enzymes in *Kluyveromyces marxianus* by promoter and signal sequence engineering

**DOI:** 10.1186/s13068-018-1232-7

**Published:** 2018-08-29

**Authors:** Jungang Zhou, Peixia Zhu, Xiaoyue Hu, Hong Lu, Yao Yu

**Affiliations:** 1 State Key Laboratory of Genetic Engineering, School of Life Sciences, Fudan University, Shanghai, 200438 China; 2 Shanghai Engineering Research Center of Industrial Microorganisms, Shanghai, 200438 China; 3 Shanghai Collaborative Innovation Center for Biomanufacturing Technology, Shanghai, 200237 China

**Keywords:** *Kluyveromyces marxianus*, Inulinase, Lignocellulolytic enzymes, Signal sequence, Promoter optimization

## Abstract

**Background:**

Taking into account its thermotolerance, high growth rate, and broad substrate spectrum, *Kluyveromyces marxianus* can be considered an ideal consolidated bioprocessing (CBP). A major obstacle to ethanol production using *K. marxianus* is the low production of lignocellulolytic enzymes, which reduces the cellulose hydrolysis and ethanol production. Thus, further improvement of enzyme expression and secretion is essential.

**Results:**

To improve the expression of lignocellulolytic enzymes, the inulinase promoter and signal sequence from *K. marxianus* was optimized through mutagenesis. A T(-361)A mutation inside the promoter, a deletion of AT-rich region inside 5′UTR (UTR∆A), and a P10L substitution in the signal sequence increased the secretory expression of the feruloyl esterase Est1E by up to sixfold. T(-361)A and UTR∆A increased the mRNA expression, while the P10L substitution extended the hydrophobic core of signal sequence and promoted secretion of mature protein. P10L and T(-361)A mutations increased secretory expressions of other types of lignocellulolytic enzymes by up to threefold, including endo-1,4-β-glucanase RuCelA, endo-1,4-β-endoxylanase Xyn-CDBFV, and endo-1,4-β-mannanase MAN330. During the fed-batch fermentation of strains carrying optimized modules, the peak activities of RuCelA, Xyn-CDBFV, MAN330, and Est1E reached 24 U/mL, 25,600 U/mL, 10,200 U/mL, and 1220 U/mL, respectively. Importantly, higher yield of enzymes by optimized promoter and signal sequence were achieved in all tested carbon sources, including the major end products of lignocellulose saccharification and fermentation, with growth on xylose resulting in the highest production.

**Conclusions:**

The engineered promoter and signal sequence presented increased secretory expressions of different lignocellulolytic enzymes in *K. marxianus* by means of various carbon resources. Activities of lignocellulolytic enzymes in fed-batch fermentation were the highest activities reported for *K. marxianus* so far. Our engineered modules are valuable in producing lignocellulolytic enzymes by *K. marxianus* and in constructing efficient CBP strains for cellulosic ethanol production.

**Electronic supplementary material:**

The online version of this article (10.1186/s13068-018-1232-7) contains supplementary material, which is available to authorized users.

## Background

Ethanol production from the lignocellulosic biomass involves four steps that include feedstock pretreatment, fractionation, enzymatic hydrolysis (saccharification), and microbial fermentation [1]. *Kluyveromyces marxianus* is a homothallic hemiascomycetous yeast species commonly isolated in dairy products, grape, and henequen [2]. It is an aerobic, Crabtree negative yeast, and generates energy from both respiratory metabolism and ethanol fermentation [3, 4]. Due to its thermotolerance, high growth rate, and the capacity to assimilate inulin, lactose, and pentose sugars like xylose and arabinose, *K. marxianus* can be considered to be a potential alternative to *Saccharomyces cerevisiae* for the production of ethanol from cellulosic feedstocks [5].

Breakdown of lignocellulose into fermentable sugars requires three major cellulase enzymes, including endo-1,4-β-glucanase (EC 3.2.1.4), cellobiohydrolase (EC 3.2.1.91), β-glucosidase (EC 3.2.1.21), and several hemicellulases, such as endo-1,4-β-xylanase (EC 3.2.1.8), endo-1,4-β-mannanase (EC 3.2.1.78), β-xylosidase (EC 3.2.1.27), and hemicellulolytic esterases [6, 7]. Enzymes for lignocellulose degradation constitute the major cost of cellulosic ethanol [8]. CBP, combining enzyme production, saccharification, and fermentation in a single step by a single microbe, was regarded as the ultimate means to lower the cost of cellulosic ethanol. Numerous attempts have been made to genetically modify natural cellulolytic bacteria such as *Cellulolytic thermophiles*, *Caldicellulosiruptor bescii*, *Thermoanaerobacterium saccharolyticum*, and *Geobacillus thermoglucosidasius*, and filamentous fungi, such as *Trichoderma* sp., *Aspergillus* sp., *Fusarium oxysporum*, and *Penicillium* sp. to produce cellulosic ethanol via CBP [9]. Several species, e.g., *C. bescii*, can directly convert cellulosic and hemicellulosic compositions to ethanol without chemical pretreatment of the feedstock [10]. However, none of these cellulolytic microbes can tolerate high concentrations of ethanol (> 10%). *S. cerevisiae* is one of the most promising microbes to develop CBP strains with cellulolytic capacity, since it exhibits both high ethanol production and tolerance [11]. An *S. cerevisiae* strain expressing both an endoglucanase D from *Clostridium cellulovorans* and a β-glucosidase from *Saccharomycopsis fibuligera* produced 9.15 g/L ethanol from 20 g/L β-glucan after 50 h of fermentation, achieving 80.3% of the theoretical ethanol yield [12]. An industrial *S. cerevisiae* strain that integrated endoglucanase, exoglucanase, and β-glucosidase into the chromosomal ribosomal DNA and delta regions can convert 63% cellulose from pretreated corn stover into ethanol with a titer of 26 g/L [13]. In addition, a *S. cerevisiae* strain engineered to use hemicellulose via coexpression of the *Trichoderma reesei* xylanase (*xyn2*), the *Aspergillus niger* xylosidase (*xlnD*), the *Scheffersomyces stipitis* xylulose kinase (*xyl3*), and *Bacteroides thetaiotaomicron* isomerase (*xylA*), produced 9 g/L of ethanol after 40 days under anaerobic fermentation [14].

*Kluyveromyces marxianus* is another CBP yeast that has attracted much attention recently with respect to its ethanol production, tolerance, and high-temperature resistance [15]. A genetically engineered *K. marxianus* strain co-displayed *T. reesei* endoglucanase and *Aspergillus aculeatus* β-glucosidase on the cell surface, produced 7.02 and 2.12 U/OD_600_ of endoglucanase and β-glucosidase, respectively, and converted β-glucan to ethanol with a yield of 4.24 g/L ethanol from 10 g/L β-glucan [16]. To improve ethanol production by *K. marxianus*, five cellulase genes, including two cellobiohydrolases, two endo-β-1,4-glucanases, a β-glucosidase gene, and a cellodextrin transporter gene from different fungi were simultaneously expressed in *K. marxianus* KY3, and the resultant strain produced 0.6 g/L ethanol from 10% avicel [17].

However, compared with separate hydrolysis and fermentation (SHF) or simultaneous saccharification and fermentation (SSF), the efficiencies of cellulose hydrolysis and ethanol production by engineered CBP *K. marxianus* strains are still low. One of the major reasons is the low production of secreted enzymes by these strains [17]. In this study, we engineered the promoter and signal sequence from *K. marxianus* inulinase-encoding gene (*INU1*) to improve the secretary expression of lignocellulolytic enzymes. Inulinase is responsible for the degradation of inulin and that supports the growth of *K. marxianus* on fructans [18]. A small fraction of extracellular inulinase is associated with the cell wall as a tetramer, and the rest extracellular inulinase is excreted into the culture fluid as a dimer [19]. In a certain *K. marxianus* strain, the latter fraction of inulinase compromises more than 60% of total secreted proteins in the supernatant [20], suggesting inulinase is expressed from a strong promoter and its secretion is initiated by an efficient signal sequence. By means of the *INU1* promoter and signal sequence, Bergkamp et al. obtained high β-galactosidase production (153 mg/L) and secretion efficiency (99%) in *K. marxianus* [21]. However, it remained to be seen how generalizable these results were to other, more economically important secreted enzymes, and if the *INU1* promoter and secretion signal sequence could be further improved.

In this study, we performed mutagenesis to increase the efficiency of the inulinase promoter and signal sequence. A mutation inside the promoter and a deletion of an A-T-rich region inside the 5′UTR increased mRNA levels and improved the secretory activity. Optimization of the signal sequence by extending the central hydrophobic region significantly improved secretion of the mature protein. The expressions of lignocellulolytic enzymes, including endo-1,4-β-mannanase, endo-1,4-β-endoxylanase, endo-1,4-β-glucanase, and feruloyl esterase, were increased by two to sixfold using optimized promoter or signal sequence. Improved expression by optimized modules can be achieved via growing in carbon sources that are the end products of lignocellulose saccharification (glucose, mannose, galactose, xylose, arabinose, and ethanol). Our study provides useful modules for high-level secretory expression of lignocellulolytic enzymes and contributes to the optimization of CBP for cellulosic ethanol production by *K. marxianus*.

## Methods

### Yeast strains

The host *K. marxianus* strain FIM-1∆U used in this study was derived from FIM-1 (China General Microbiological Culture Collection Center, CGMCC No. 10621). Genomic DNA was extracted from FIM-1 by Yeast Genomic DNA Extraction Kit (D1900, Solarbio, Beijing, China). The *URA3* gene, including 703 bp promoter, 804 bp ORF and 488 bp terminator, was amplified from the genomic DNA of FIM-1 by means of Phanta Max Super-Fidelity DNA Polymerase (P505, Vazyme, Nanjing, China) and primer pair URA3-F/URA3-R. 3′ adenine overhangs were added by Taq polymerase (E001, Novoprotein, Shanghai, China). The purified PCR product was ligated into the pMD18-T vector (6011, Takara, Dalian, China) to obtain pZP12. The *URA3* ORF in pZP12 was removed by PCR mutagenesis to obtain pZP13. Briefly, pZP12 was amplified by Phanta Max Super-Fidelity DNA Polymerase with primer pair URA3∆-F/URA3∆-R. After 18 cycles of PCR, *Dpn*I (1609, Takara) was added into the PCR mixture to digest the methylated template. *Dpn*I-treated product was transformed into *Escherichia coli* DH5α and correct transformant was named as pZP13. The *URA3* deletion cassette was amplified from pZP13 by Phanta Max Super-Fidelity DNA Polymerase with primer pair URA3-F/URA3-R. 1 μg purified PCR product was transform into FIM-1 by lithium acetate method as described before [22]. Transformants were selected on YPD + 5FOA plate (2% glucose, 2% polypeptone, 1% yeast extract, 2% agar, 0.1% 5-fluoroorotic acid) and identified by PCR using primer pair URA3-F/URA3-R2. The correct transformant was given the strain ID as FIM-1∆U. Cells were grown at 30 °C. Plasmid used in the construction are listed in Additional file 1: Table S1 and primers are listed in Additional file 2: Table S2.

### Plasmids

All plasmids used in this study are listed in Additional file 1: Table S1). *INU1* gene, including 1136 bp promoter, 1671 bp ORF and 865 bp terminator, was amplified by Phanta Max Super-Fidelity DNA Polymerase and primer pair INU1-F/INU1-R. The same polymerase was used in the PCR reactions described below. 3′ adenine overhangs were added by Taq polymerase. Purified PCR product was ligated with pMD18-T vector to obtain pZP14. 79–1668 bp of *INU1* ORF in pZP14 was removed, and multiple cloning sites (MCS) were introduced by PCR mutagenesis with primer pair MCS-F/MCS-R as described above. Resultant plasmid was called pZP15. A fragment containing the *URA3* gene, pBluescript II KS(+) vector part, and autoreplicating KD element was amplified from pUKD [20], by means of primer pair PUKD-F/PUKD-R. Another fragment containing inulinase promoter, inulinase signal peptide, MCS, and inulinase terminator was amplified from pZP15 by primer pair INU1-F/INU1-R. The two fragments were ligated together by Gibson assembly master mix (E2611S, NEB, USA) and the resultant plasmid is called pUKDN132. pUKDN132 served as a backbone for expressing heterologous proteins (Additional file 3: Figure S1). *Est1E* (Genbank: MH212232) was amplified from metagenome of yak rumen with primer pair Est1E-F/Est1E-R. Purified PCR product of *Est1E* was inserted between *Spe*I and *Not*I sites of pUKDN132 to obtain pZP17. M1, M2, and M3 promoters were amplified from pZP17 by means of primer pairs M1-F/MP-R, M2-F/MP-R, and M3-F/MP-R, respectively, and were inserted between *Sac*II and *Sma*I sites of pZP17 to obtain pZP18–pZP20. UTR∆A mutation were introduced into pZP17 to obtain pZP22 by PCR mutagenesis using primer pairs UTR∆A-F/UTR∆A-R. A(-1104)T, A(-566)T, T(-351)A, A(-266)G, T(-233)A, or P10L mutation was introduced into pZP17 by PCR mutagenesis using primer pairs A(-1104)TF/A(-1104)TR, A(-566)T-F/A(-566)T-R, T(-351)A-F/T(-351)A-R, A(-266)G-F/A(-266)G-R, T(-233)A-F/T(-233)A-R, or P10L-F/P10L-R, and resultant plasmids were named as pZP23, pZP24, pZP25, pZP26, pZP27, or pZP28. UTR∆A or P10L mutation was introduced into pZP25, respectively, by PCR mutagenesis using primer pair UTR∆A-F/UTR∆A-R or P10L-F/P10L-R, and the resultant plasmids were named as pZP29 or pZP30, respecively. P10L mutation was introduced into pZP29 by PCR mutagenesis using primer pair P10L-F/P10L-R, and the resultant plasmid was named as pZP31. His_6_-tagged *Est1E* was amplified from pZP17 by primer pair Est1E-F/His6-R and inserted between *Spe*I and *Not*I sites of pUKDN132 or pZP28 to obtain pZP32 or pZP33. P10I, P10G, P10S, P10K, or P10D substitutions inside inulinase signal sequence of pZP17 were performed by PCR mutagenesis with primer pairs P10I-F/P10I-R, P10G-F/P10G-R, P10S-F/P10S-R, P10K-F/P10K-R, or P10D-F/P10D-R to obtain plasmid pZP34, pZP35, pZP36, pZP37, or pZP38. Liner double-strand DNA containing inulinase signal sequence from *K. lactis* was synthesized by Genewiz (Suzhou, China) and named as KLSS. KLSS was used as a megaprimer to replace inulinase signal sequence of *K. marxianus* in pZP17 by mutagenesis PCR, and the resultant plasmid was named pZP39. P10L mutation was introduced into inulinase signal sequence of pZP39 by PCR mutagenesis using primer pairs KL-P10L-F/KL-P10L-R to obtain pZP40. *MAN330* [23], a truncated form of *manA* (Genbank: AY534912) was amplified by primer pair Man-F/Man-R, and inserted between *Spe*I and *Not*I sites of pUKDN132, pZP25, pZP22, or pZP28 to obtain pZP41, pZP42, pZP43, or pZP44. *Xyn*-*CDBFV* [24], a thermostable mutant of *xynC* (Genbank: AF123252) was amplified by Xyn-F/Xyn-R, and inserted between *Spe*I and *Not*I sites of pUKDN132, pZP25, pZP22, or pZP28 to obtain pZP45, pZP46, pZP47, or pZP48. *RuCel1A* (Genbank: GU132859) was amplified by CelA-F/CelA-R [25], and inserted between *Spe*I and *Not*I sites of pUKDN132, pZP25, pZP22, or pZP28 to obtain pZP49, pZP50, pZP51, or pZP52. Primers used in the plasmid construction are listed in Additional file 2: Table S2.

### Random mutagenesis of *INU1* promoter and signal sequence

Random mutagenesis of the *INU1* promoter and signal sequence was conducted by MEGAWHOP Cloning [26]. Briefly, the *INU1 p*romoter and signal sequence were amplified from pZP17 by error-prone PCR using the GeneMorph^®^ II Random Mutagenesis Kit (200550, Agilent Technologies) with primer pair MutP-F/MutP-R. The PCR product was purified, and used as a megaprimer to amplify pZP17 by Phanta Max Super-Fidelity DNA Polymerase. At the end of PCR, *Dpn*I was added into the PCR mixture to digest the methylated template. *Dpn*I-treated product was transformed into FIM-1∆U by lithium acetate method [22]. Transformants were selected on SD plate (0.67% yeast nitrogen base without amino acids, 2% glucose, 2% agar) and subjected to the screen described below.

### Screen for mutants expressing high levels of Est1E

6500 transformants containing mutant plasmids were subjected to the following screen. Transformants were inoculated into 600 μL YG medium (2% yeast extract, 4% glucose) in 24-well microplates and cultured for 72 h. Plates were centrifuged at 3000 rpm for 5 min, and supernatant was collected. The activity of Est1E in the supernatant was measured by means of 2-chloro-4-nitrophenyl ferulate (CNPF) as substrate [27]. CNPF was diluted to 1 mM by PBST (137 mM NaCl, 2.7 mM KCl, 10 mM Na_2_HPO_4_, 1.8 mM KH_2_PO_4_, 2.5% Triton X-10, pH 6.4). 20 μL supernatant was mixed with 180 μL CNPF solution and incubated at 37° for 20 min. The release of 2-chloro-4-nitrophenol (CNP) was measured by the absorbance at 410 nm. One unit (U) of feruloyl esterase activity was defined as 1 nmol of CNP from the substrate per minute. Plasmids were extracted from the transformants expressing high level of Est1E by Yeast Plasmid Extraction Kit (D1160, Solarbio) and were transformed into *E. coli*. Plasmids recovered from *E. coli* were transformed back into FIM-1∆U by the lithium acetate method. Transformants were cultured in 50 mL YG for 72 h, and enzymatic activities in the supernatant were measured as above. Plasmids that drove high-level expression of Est1E were Sanger sequenced to identify mutations inside the inulinase promoter and signal sequence.

### Analysis of plasmid copy numbers and transcriptional analysis

pZP17, pZP22, pZP25, pZP28 were transformed into FIM-1∆U. Transformants were grown in SD liquid medium (0.67% yeast nitrogen base without amino acids, 2% glucose) overnight. Cells were diluted into YG medium to start at an OD_600_ of 0.2. 1 × 10^8^ cells were collected after 6 h. Genomic DNA was prepared by Yeast Genomic DNA Extraction Kit. qPCR was performed by means of SYBR Premix Ex TaqII (RR820A, Takara) in a LightCycler 480 II Real-Time PCR System (Roche Applied Science, Penzberg, Upper Bavaria, Germany). Plasmid copy numbers were determined by comparing the level of *Est1E* to that of endogenous *LEU2*. For transcriptional analysis, transformants were grown in SD liquid medium overnight. Cells were diluted into YG or YI medium (2% yeast extract, 4% inulin) to start at an OD_600_ of 0.2. 1 × 10^8^ cells were collected after 3, 6 and 9 h. Total RNA was extracted using ZR Fungal/Bacterial RNA MiniPrep (R2014, Zymoresearch, California, USA) and reverse transcribed into cDNA by PrimeScript RT (RR037A, Takara). qPCR was performed as above, and primers used are listed in Additional file 2: Table S2.

### Whole cell extract and Western blot assay

pZP32 and pZP33 were transformed into FIM-1∆U. Transformants were grown in YG medium and 3 × 10^8^ cells were collected after indicated times. Cells were washed and resuspended in 400 μL lysis buffer (50 mM HEPES (pH 7.5), 140 mM NaCl, 1 mM EDTA, 1% Triton X-100, 0.1% Na-Deoxycholate) supplemented with protease inhibitors cocktail (05892970001, Roche Applied Science). Cells were mixed with 400 μL acid-washed glass beads (G8772, Sigma-Aldrich, Missouri, USA) and processed by a bead-beater (FastPrep-24, MP, California, USA) at 6 m/s for 2 min. Lysate was centrifuged at 13,200 rpm for 20 min at 4°. 100 μL Supernatant was supplemented with 25 μL 5XSDS PAGE loading buffer (150 mM Tris–HCl (pH 7.0), 12% SDS, 6% 2-mercaptoethanol, 30% glycerol (V/V), 0.05% Coomassie Brilliant Blue G-250) and boiled. 10 μL Samples were subjected to Western blot assay [28]. Anti-His Tag antibody (1:5000 dilution) (M30111, Abmart, Shanghai, China), anti-histone H3.1 antibody (1:3000 dilution) (P30266, Abmart) and horseradish peroxidase conjugated goat-anti-mouse secondary antibody (1:3000 dilution) (074–1806, KPL, USA) were used in the Western blot. The blots were visualized by ECL prime Western blotting detection reagent (RPN2232, GE Healthcare, Illinois, USA) and scanned by GeneGnome HR system (Syngene, Cambridge, UK).

### Enzymatic assays

pZP17–pZP31 and pZP34–pZP40 were transformed into FIM-1∆U separately for expressing Est1E, pZP41–pZP44 for expressing MAN330, pZP45–pZP48 for expressing Xyn-CDBFV, and pZP49–pZP52 for expressing RuCelA. Transformants were selected on SD plates. Transformants were grown in 50 mL YG for 72 h, and supernatant was collected for enzymatic assay. The activity of Est1E was measured as described above. Activity of MAN330 was assayed by means of 0.5% locust bean gum (G0753, Sigma-Aldrich, USA) in 50 mM glycine–NaOH buffer (pH 9.5) at 68° [23]. The activity of Xyn-CDBFV was assayed in 50 mM acetate buffer (pH 5.5) containing 1% wheat arabinoxylan (P-WAXYL, Megazyme, Bray, Ireland) at 68° [29], while the activity of RuCelA was assayed in 50 mM acetate buffer (pH 5.5) containing 1% carboxymethyl cellulose sodium (C5678, Sigma-Aldrich, USA) at 50° [25]. One unit (U) of activity of MAN330, Xyn-CDBFV, or RuCelA was defined as the amount of enzyme releasing 1 µmol of reducing sugar per minute.

### Fed-batch fermentation

Seed culture was prepared by growing transformants in SD liquid medium for 16 h. Fed-batch fermentations were carried out in a 5 L bioreactor (BXBIO, Shanghai, China) equipped with controllers for pH, temperature, agitation, and dissolved oxygen. The temperature was maintained at 30°, and pH value was controlled automatically to 5.5 with the ammonium hydroxide during the fed-batch fermentation. The dissolved oxygen concentration maintained above 10% of air saturation automatically varied with the agitation speed at the fixed air rate 3 L/min. The recipe of synthetic medium used in the fermentation was based on a previous one, in which sucrose was replaced by 1% glucose [30]. The fed-batch fermentation started by the addition of a 10% inoculum. After depletion of glucose, a concentrated medium containing 60% glucose was pumped into the reactor with flow rates between 20 and 35 mL/h. Cell growth was monitored by the cell density (OD_600_ nm) and wet cell weight. Supernatant collected at indicated time points was subjected to enzymatic assays as described above.

### Growth in various carbon sources

Medium was prepared with 2% of yeast extract and 4% (w/v) of carbon source: inulin, glucose, cellobiose, xylose, l-arabinose, mannose, galactose, or ethanol. Transformants were grown in the indicated medium for 72 h, and the supernatant was collected and subjected to enzymatic assays as described above.

## Results

### Characterization of mutations that improve secreted protein production

**Fig. 1:**
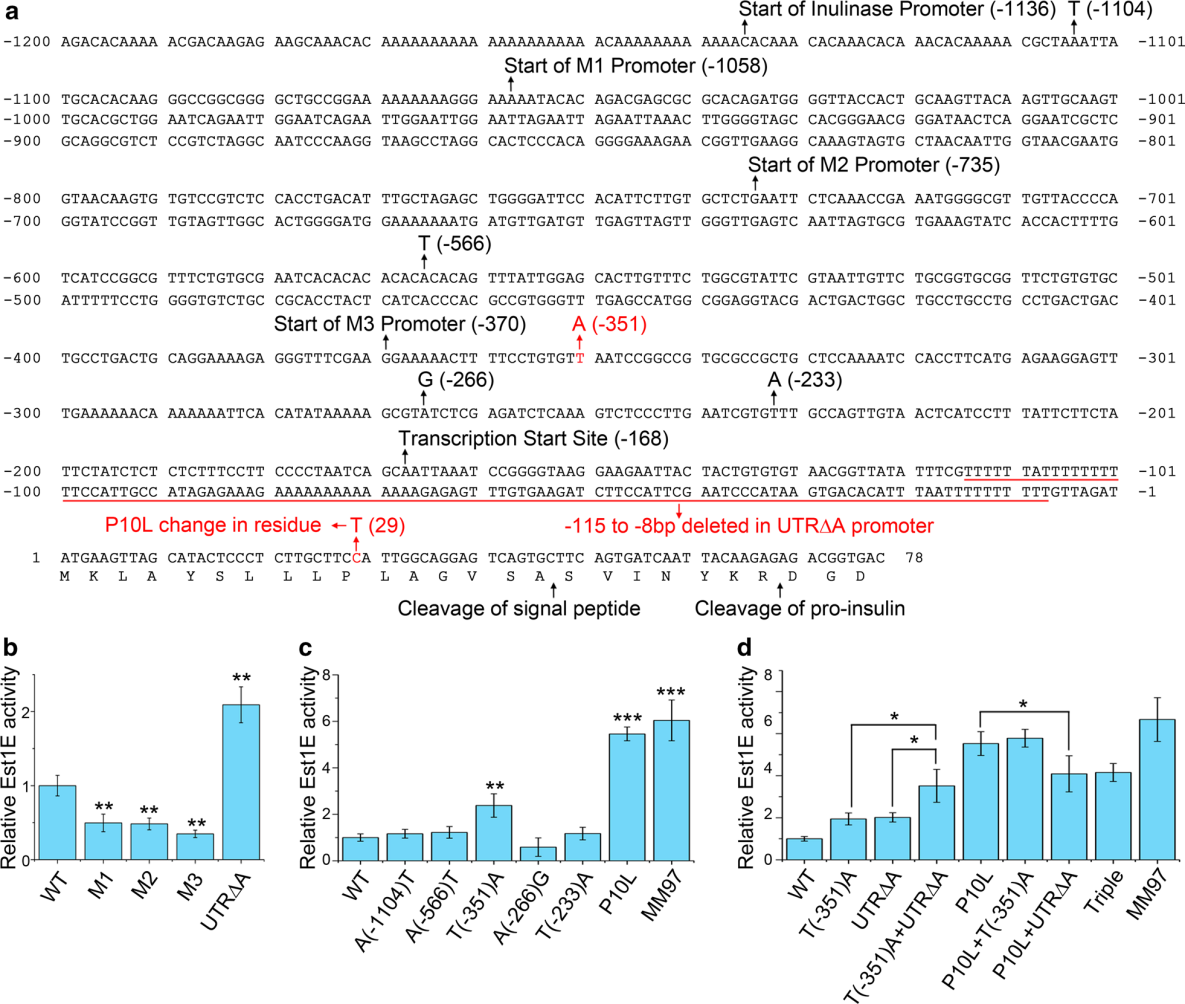
The sequence of the inulinase promoter and signal sequence, with mutations annotated. **a** Map of inulinase promoter and signal sequence. Mutants that improved expression of the reporter gene are in red. **b** Relative Est1E activity expressed by the WT or mutant promoter. The expression of a reporter gene, *Est1E*, was driven by WT or the mutant promoter on a plasmid. The activity of Est1E expressed from the WT promoter was designated as unit 1. Values in (**b**) and below represent mean ± S.D. from four parallel cultures. (*p < 0.05, **p < 0.01, ***p < 0.001). **c** Relative Est1E activity expressed by the inulinase promoter containing point mutation. **d** Relative Est1E activity expressed by double or triple mutant

The longest inulinase promoter characterized so far started from 1053 bp upstream of the start codon [31], which is corresponding to − 1058 bp in the strain used in this study. Slight shift of position is due to variations of different *K. marxianus* strains. To explore potential regions that contribute to the activity of the inulinase promoter, we used a region starting at 1136 bp 5′ of the start codon (Fig. 1a). This promoter, together with 1–78 bp of the inulinase ORF, was cloned into a plasmid to drive the expression of *Est1E*, a reporter gene encoding feruloyl esterase. 1–78 bp encodes N-terminal 26 aa of inulinase, including 16 aa of signal peptide, 7 following residues chopped by KEX2-like endoprotease, and 3 extra residues kept to ensure the efficient cleavage (Fig. 1a) [21].

The activity of short promoters reported previously was measured for comparison. Short promoters included those started at − 1058 bp (M1 promoter), − 735 bp (M2 promoter), and − 370 bp (M3 promoter). M1 promoter corresponded to − 1053 bp promoter, and M3 promoter to − 353 bp promoter identified by Gao et al. [31]. The M3 promoter excluded a potential Mig1-binding site and exhibited higher strength than the M1 promoter. The M2 promoter corresponding to − 730 bp promoter identified by Bergkamp et al. was applied to express β-galactosidase, glucose oxidase, and thermophilic esterase [21, 32, 33]. The activity of a promoter starting at − 1136 bp was at least twice as high as that of the M1, M2, or M3 promoters (Fig. 1b), suggesting that the region between − 1136 and − 1058 bp is required for the full activity of the inulinase promoter. A promoter starting at − 1136 bp served as the wild-type (WT) promoter in this following study. Previous work has shown that GC content of 5′ UTR contributes to the mRNA stability [34]. Deletion of the AT-rich region inside 5′ UTR increased the GC content in 5′UTR, and that led to a twofold increase in enzymatic activity (Fig. 1b).

Using random mutagenesis and screening for increased Est1E activity, we identified a mutant MM97 with a 6-site mutation in the *INU1* promoter and signal sequence that increased Est1E activity sixfold. Subsequently, single point mutants were constructed separately to evaluate their contributions to the improvement. As shown in Fig. 1c, A(-1104)T, A(-566)T, A(-266)G, or T(-233)A mutation did not improve the activity of the promoter. The T(-351)A mutation doubled the activity of Est1E, and a substitution of proline at position 10 by leucine (P10L) increased the activity by fivefold, suggesting P10L contributes most of the improvement observed in MM97 mutant. Relationships among the T(-351)A, UTR∆A and P10L mutations were investigated next (Fig. 1d). Combination of T(-351)A and UTR∆A mutation displayed an additive effect, which led to a threefold increase in activity. This result suggests two mutations improve the promoter activity in different pathways. Combination of T(-351)A and P10L did not increased the activity of P10L mutant, suggesting that P10L bypasses the improvement from T(-351)A. Combination of UTR∆A with P10L significantly reduced the activity comparing to P10L, suggesting these two mutations are not compatible to improve the secretory expression. A triple mutant containing all three mutations displayed the same level of activity as double mutant containing UTR∆A and P10L. This suggests the T(-351)A mutation can’t rescue the incompatibility between UTR∆A and P10L.

### Transcriptional analysis of cells carrying T(-351)A, UTR∆A or P10L plasmid

**Fig. 2:**
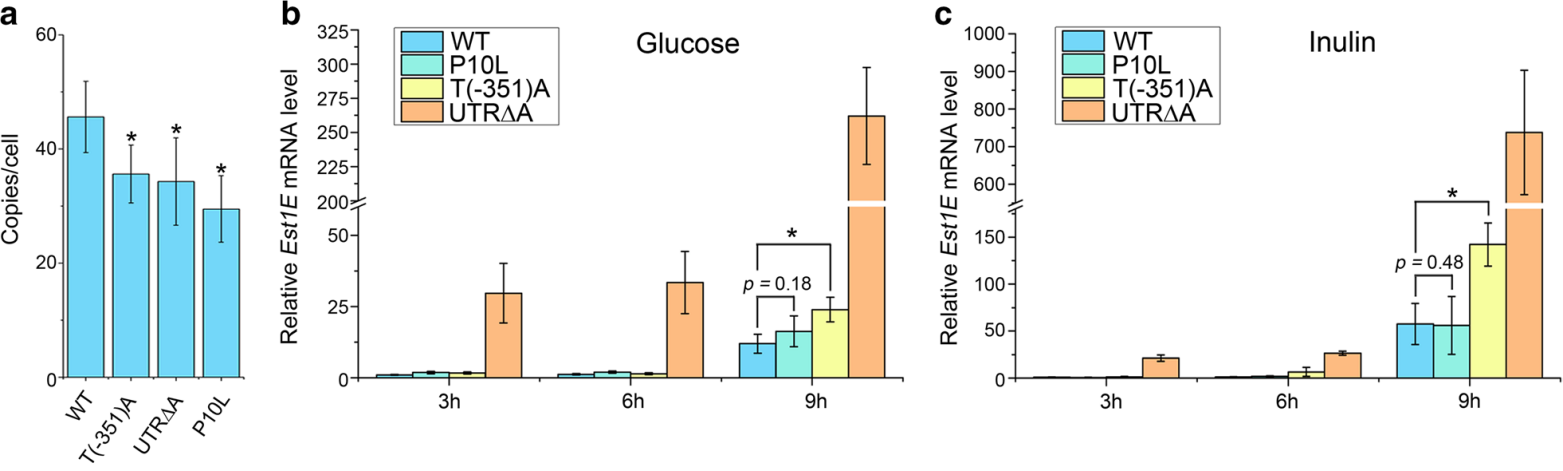
Transcriptional analysis of cells carrying T(-351)A, UTR∆A, or P10L plasmid. **a** Copies of WT or mutant plasmid. Cells containing WT, T(-351)A, UTR∆A, or P10L plasmid were grown in YG medium until exponential stage. Copy number was calculated relative to that of endogenous *LEU2*. Values represent mean ± S.D. from four parallel cultures. (*p < 0.05) **b**, **c** Relative mRNA level of *Est1E* expressed by WT or mutant plasmid in YG (**b**) or YI medium (**c**). mRNA level of *Est1E* was calculated relative to 18s rDNA and relative level of *Est1E* in cells containing WT plasmid at 3 h is designated as unit 1. Values represent mean ± S.D. from three parallel cultures

To investigate the mechanism underlying the improvement of secretory activity, copy numbers of plasmids were analyzed. We found the plasmid copy number was inversely correlated with expression. All higher expressing mutants has significantly reduced plasmid copy number compared to WT (Fig. 2a). Thus, improved activity observed in the mutants was not due to the increase of plasmid copy number.

We found no significant difference in *Est1E* mRNA levels between cells carrying WT and those carrying P10L plasmid when cells were grown in a medium containing glucose or inulin (Fig. 2b, c). Thus, the P10L mutation in the signal sequence does not affect *Est1E* mRNA levels. The level of *Est1E* mRNA expressed from T(-351)A plasmid was doubled in glucose medium and increased by threefold in inulin medium, comparing that expressed from the wild-type plasmid. Since the T(-351)A mutation resides 183 bp upstream of the transcription start site, T(-351)A might increase the amount of mRNA by promoting transcriptional initiation [35]. The T(-351)A mutation is in the middle of a putative InuR-binding sites (CGGATTAACACAGG, complementary strand). InuR is a Zn(II)_2_Cys_6_ transcriptional activator involved in the regulation of inulinolytic genes in *A. niger* [36]. No InuR-like factor has been identified in *K. marxianus* so far. Whether the T(-351)A mutation increases the affinity of the promoter to a transcriptional activator requires further study.

The UTR∆A mutation caused a 22-fold increase in mRNA level in glucose medium and a 13-fold increase in inulin medium (Fig. 2b, c). A previous study showed that a mutation in the 5′UTR increased stability of mRNA [37], which might be true in this case. However, only a twofold increase of Est1E activity was observed in the UTR∆A mutant (Fig. 1b). The 5′UTR is well known to be implicated in the regulation of translation [38]. Therefore, a twofold increase of enzymatic activity from the UTR∆A mutation is likely due to a net outcomes of increased mRNA stability and reduced translation efficiency.

### The P10L substitution in signal peptide improves secretion of mature protein

**Fig. 3:**
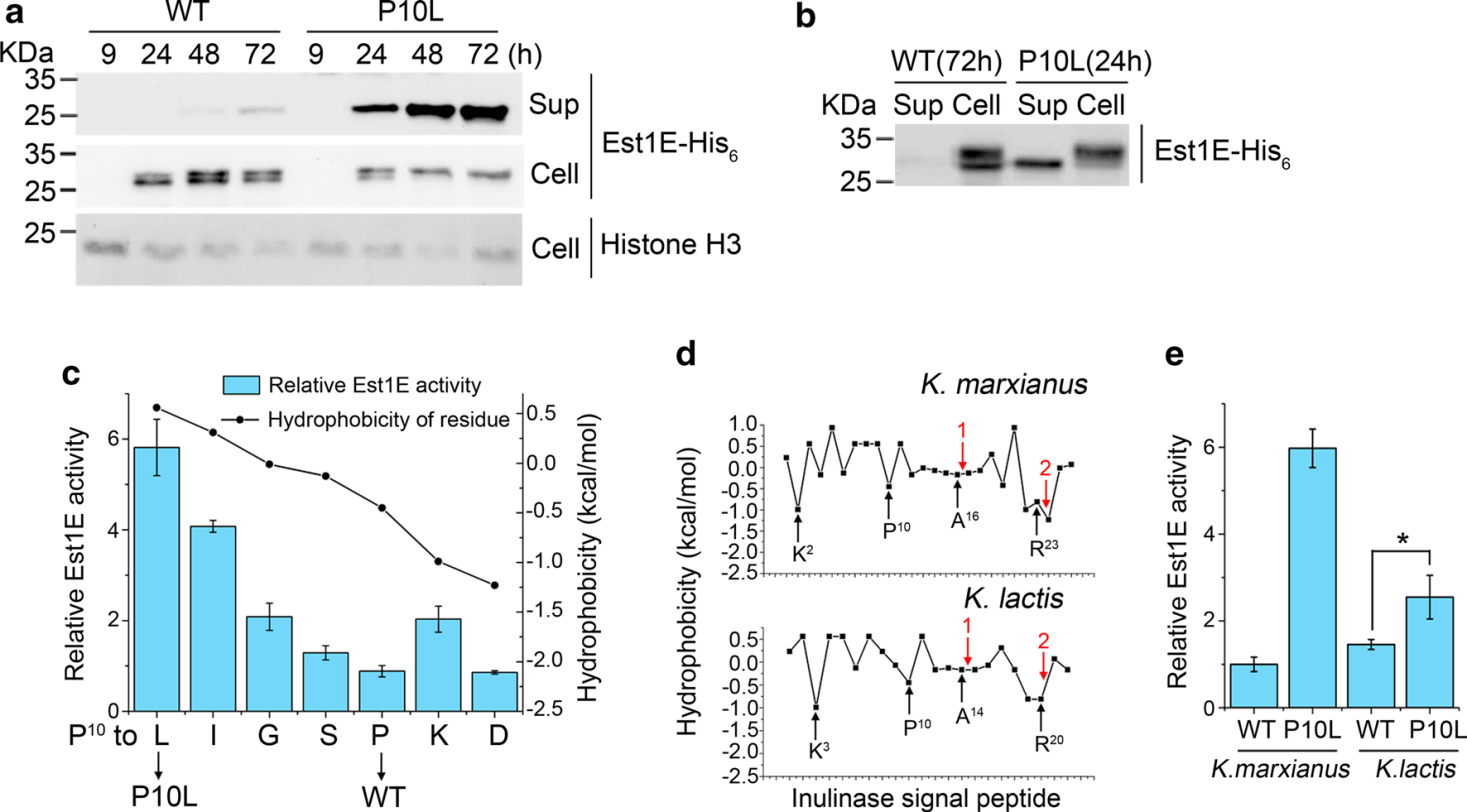
Effect on secretion by substituting proline^10^ inside inulinase signal sequence. **a** Western blot assay to examine the level of Est1E-His_6_ in cell lysate (Cell) and supernatant (Sup). **b** Comparison of Est1E-His_6_ in supernatant and cell lysate. **c** Relative Est1E activity in mutant containing various substitutions of proline^10^. Activity of Est1E expressed by WT signal sequence is designated as unit 1. Values in (**c**) and below represent mean ± S.D. from four parallel cultures. Hydrophobicity of residue was represented by Δ*G* of transfer from phosphatidylcholine interface to water [53]. **d** Hydrophobicity of residues inside inulinase signal sequence from *K. marxianus* and *K. lactis*. The cleavage site of signal peptide (1) and that of pro-insulin (2) are indicated by red arrows. Cleavage sites in *K. lactis* were predicted by SignalP 4.0 [54]. **e** Relative activity of Est1E expressed by P10L signal sequence from *K. marxianus* and *K. lactis*. Activity of Est1E expressed by WT inulinase signal sequence from *K. marxianus* is designated as unit 1 (*p < 0.05)

Since P10L is located in the middle of the signal peptide, the effect of P10L on the secretion of Est1E was investigated. After growing in the YG medium for 24 h, Est1E could be clearly detected in the supernatant from cells containing the P10L plasmid. The level of Est1E-His_6_ kept climbing till 72 h. In contrast, very little Est1E could be observed in the supernatant from cells containing WT plasmid even after 72 h (Fig. 3a). The amount of Est1E in the cell lysate gave opposite results. There was less Est1E retained in the cells containing P10L plasmid than that in the cells containing WT plasmid (Fig. 3a). The result indicates that P10L substitution strongly promotes the secretion of Est1E.

It is noticeable that two nearby bands were detected in the cell lysate (Fig. 3a). In the cells containing WT plasmid, both bands were observed between 24 and 72 h. In the cells containing P10L plasmid, the upper bands were observed between 24 and 72 h, while the lower bands were only visible at 24 h and then disappeared at 48 h and 72 h. By loading selected samples from supernatant and cell lysate side by side, it was obvious that the size of the lower bands was the same as that of bands in supernatant (Fig. 3b). This result suggests that the lower bands in the cell lysate correspond to the mature Est1E after cleavage by signal peptidase and KEX2-like endoprotease. The upper bands probably represent the pre-Est1E before cleavage. The P10L mutation substantially reduced the amount of cleaved Est1E in the cells. It suggests P10L substitution promotes proper cleavage of signal peptide, since improper cleavage of signal peptide could cause intracellular retaining of cleaved proteins [39].

Leucine is more hydrophobic than proline. To investigate the importance of hydrophobicity at position 10, proline was substituted to various residues with different hydrophobicity. With an exception of lysine, the more hydrophobic the residue at position 10, the higher activity of Est1E in the supernatant (Fig. 3c). We analyzed the hydrophobicity of residues in inulinase signal sequences from *K. marxianus* and *K. lactis*, and found both signal sequences shared very similar pattern of hydrophobicity (Fig. 3d). A typical signal sequence consists of an N-terminal hydrophilic n-region, a central hydrophobic h-region, and a C-terminal hydrophilic c-region. The n-region usually contains a basic residue [40]. In the inulinase signal sequence, the N terminal basic residue is a lysine at position 2 in *K. marxianus*, or at position 3 in *K. lactis*. A stretch of hydrophobic residues following that lysine are present in both inulinase signal sequence, which composes the h-region. The c-region often contains helix breaking proline and small uncharged residues that determine the site of signal peptide cleavage [40]. Thus, a proline at position 10 probably marks the start of the c-region. Substituting proline 10 into a more hydrophobic residue like leucine extended the hydrophobic core, and that might promote recognition by receptor proteins, such as SRP and translocon. Meanwhile, shortened c-region might also change the efficiency of cleavage. To validate whether P10L substitution in *K. lactis* improves secretion, signal sequence of *K. marxianus* on the plasmid was replaced by WT or P10L signal sequence from *K. lactis*. Est1E expressed from WT inulinase signal sequence from *K. lactis* was slightly higher than that from *K. marxianus*, suggesting efficiencies of inulinase signal sequences of *Kluyveromyces* are close. Interestingly, P10L substitution in the signal sequence from *K. lactis* improved the secretory activity by twofold comparing to WT sequence (Fig. 3e). Thus, extension of the hydrophobic core of signal sequence might be a conserved way to improve secretion initiated by the inulinase signal peptide.

### T(-351)A, UTR∆A and P10L improve secretory expression of lignocellulolytic enzymes

**Fig. 4:**
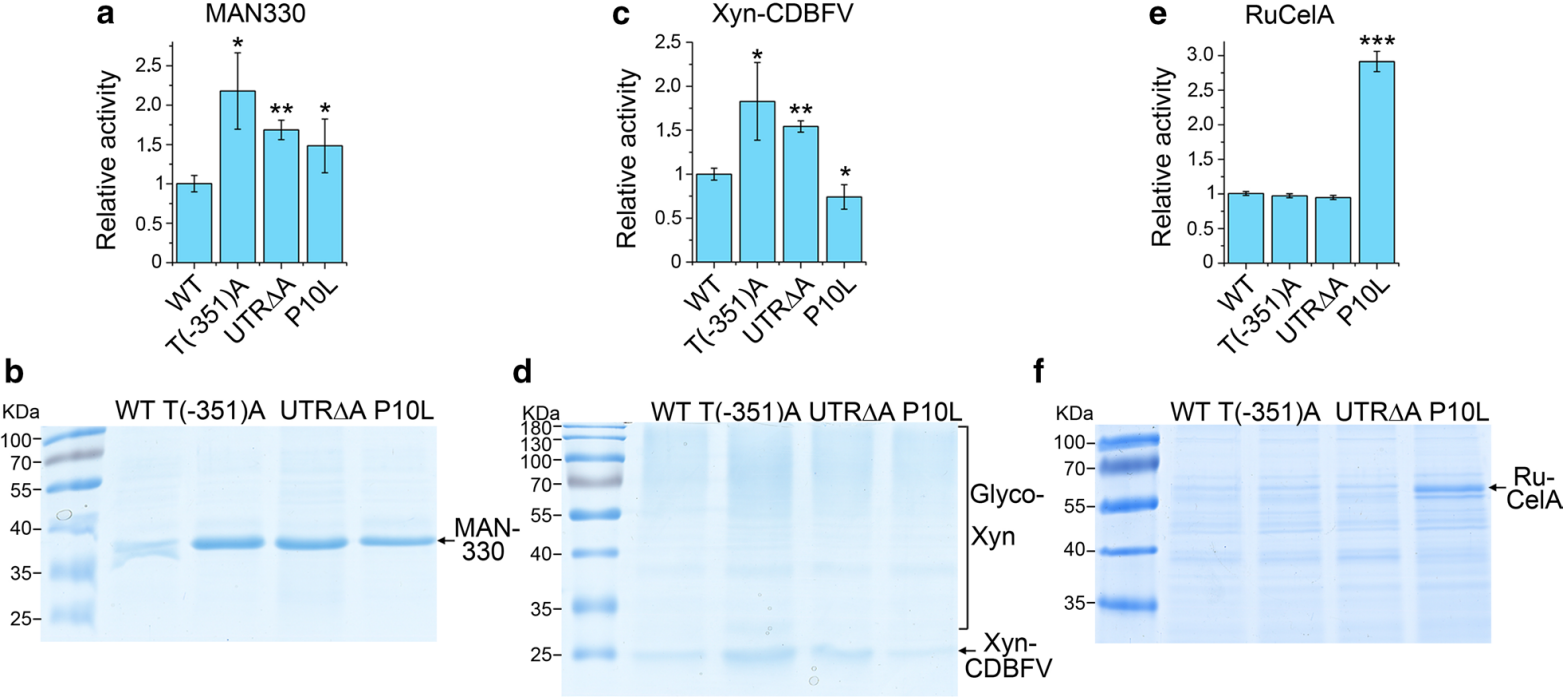
Secretory expression of lignocellulolytic enzymes by WT or mutant plasmid. Activity of MAN330 (**a**), Xyn-CDBFV (**c**) or RuCelA (**e**) is indicated. Cells containing WT or mutant plasmid were grown in YG medium for 72 h, and activity in the supernatant was measured. Activity expressed by WT plasmid was designated as unit 1. Values represent mean ± S.D. from three parallel cultures. (*p < 0.05). SDS-PAGE of supernatant from representative cultures was shown in (**b**, **d**, **f**)

Endoglucanase, β-1,4-xylanase, and β-1,4-mannanase initiate the degradation of lignocellulose by attacking the β-1,4-bond of cellulose and hemicellulose [6, 7]. A β-1,4-endoglucanase RuCel1A, a β-1,4-endoxylanase Xyn-CDBFV and a truncated β-1,4-endomannanase MAN330 were incorporated to investigate the effect of T(-351)A, UTR∆A or P10L mutation on the secretory expression of different enzymes. Compared to the WT plasmid, T(-351)A, UTR∆A and P10L mutation improved the secretory activity of MAN330 by 2.2-, 1.7- and 1.5-fold, respectively (Fig. 4a). Increased amount of MAN330 in the supernatant was visible by SDS-PAGE (Fig. 4b). T(-351)A and UTR∆A mutations improved the secretory activity of Xyn-CDBFV by 1.8- and 1.5-fold, respectively. However, the activity of Xyn-CDBFV in the cells containing the P10L plasmid was even lower than that containing the WT plasmid (Fig. 4c, d). Although T(-351)A and UTR∆A mutation could not improve the secretory activity of RuCel1A, P10L significantly improved the activity by threefold (Fig. 4e, f). In sum, the secretory expression of three different lignocellulosic enzymes could be improved by at least one of three mutants. It is likely to achieve improved secretory expression of other heterologous proteins by choosing the specific mutant plasmid(s) described in this study.

### High-level expression of lignocellulolytic enzymes in fed-batch fermentation by *K. marxianus*

The filamentous fungi, *T. reesei* and *A. niger*, are the main industrial producers of lignocellulolytic enzymes, and a long special induction and culture is the limiting step of traditional enzyme production [17]. To evaluate the industrial potential of fast-growing *K. marxianus* in producing lignocellulolytic enzymes, cells carrying WT plasmid or representative mutant plasmids were cultured in a 5-L reactor containing synthetic medium, the latter being chosen to reduce cost. After 72-h fed-batch fermentation, the wet cell mass of all cells reached more than 400 g/L.

**Fig. 5:**
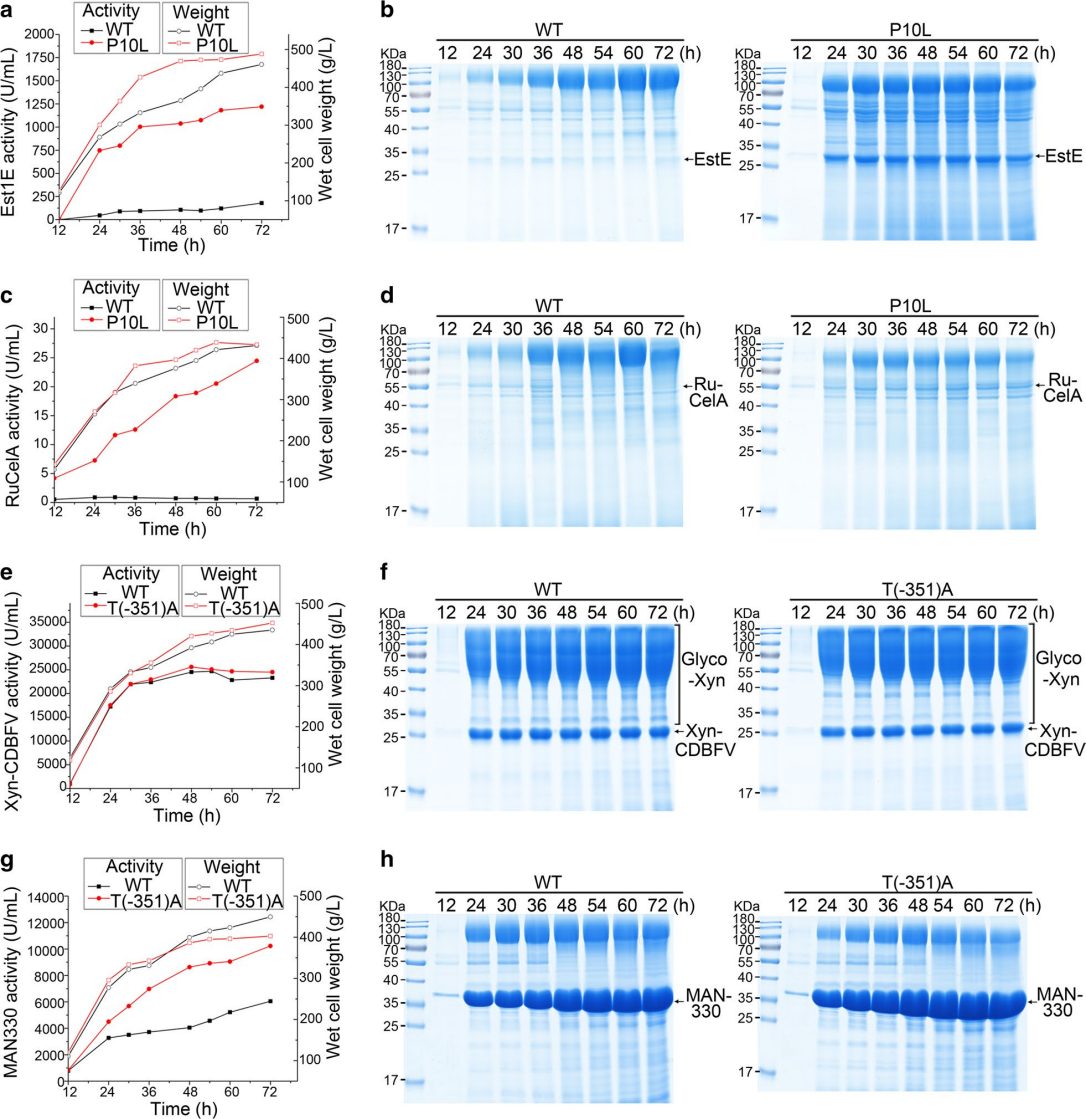
Expression of lignocellulolytic enzymes by WT or mutant plasmids in fermentor. Cells containing WT or mutant plasmid were grown in a 5 L fermentor for 72 h. Curves of enzymatic activity and wet cell weight were plotted for cells expressing Est1E (**a**), RuCelA (**c**), Xyn-CDBFV (**e**) or MAN330 (**g**). SDS-PAGE of the supernatant at different time points is shown in (**b**, **d**, **f**, **h**)

Feruloyl esterase produced by the P10L plasmid accumulated rapidly at early stage, and the activity reached 750 U/mL after 24 h. The increase of activity slowed down after that. The feruloyl esterase activity produced by the P10L plasmid peaked after 72 h, reaching 1220 U/mL, which was about seven times of that expressed by WT plasmid (Fig. 5a). The results were consistent with earlier estimates in flasks. The theoretical molecular weight of Est1E is 29 kDa. As shown in the SDS PAGE (Fig. 5b), Est1E was expressed at expected size. Calculated by the specific activity of Est1E (2400 U/mg) (Additional file 4: Figure S2), the protein concentration of Est1E expressed by P10L plasmid reached 0.5 g/L. Endo-β-glucanase RuCelA produced by P10L plasmid increased linearly during the fed-batch fermentation. The activity of RuCelA produced by the P10L plasmid reached 24 U/m after 72 h, which is 36 times of that produced by WT plasmid (Fig. 5c). The theoretical molecular weight of RuCelA is 62 kDa, and RuCelA was expressed at expected size (Fig. 5d). Calculated by the specific activity of RuCelA (65 U/mg) (Additional file 5: Figure S3), the protein concentration of RuCelA expressed by P10L plasmid was 0.37 g/L. Thus, signal sequence optimization is effective in the large-scale expressions of Est1E and RuCelA.

In fed-batch fermentation, the T(-351)A mutation showed no distinct effect on endo-β-xylanase production (Fig. 5e). The growth and activity curves of cells carrying WT and mutant plasmid were similar. The highest activities of Xyn-CDBFV were achieved at 48 h, when activity expressed by T(-351)A plasmid was 25,600 U/mL and that by WT plasmid was 24,500 U/mL Calculated by the specific activity of Xyn-CDBFV (4000 U/mg) [29], the protein concentration of Xyn-CDBFV expressed by T(-351)A plasmid was 6.4 g/L. The theoretical molecular weight of Xyn-CDBFV is 25 kDa. Although a band of expected size was observed in SDS-PAGE, it was noticeable that a smear at high molecular weight was produced during fermentation (Fig. 5f). After deglycosylation, the smear disappeared, and one thicker band was observed at the expected size (Additional file 6: Figure S4), indicating the smear is the hyperglycosylated form of Xyn-CDBFV. Since N-hypermannose glycosylation inhibits secretion of cellulases in *S. cerevisiae* [41], hyperglycosylation of Xyn-CDBFV might bypass the improvement by T(-351)A mutation. In contrast, improved expression of β-1,4-endomannanase by the T(-351)A plasmid was reproduced in large-scale fermentation. After 72 h, the activities of MAN330 produced by T(-351)A and WT plasmid reached 10,200 U/mL and 6000 U/mL, respectively (Fig. 5g). The theoretical molecular weight of MAN330 is 35 kDa and MAN330 was expressed at expected size (Fig. 5h). Calculated by the specific activity of MAN330 (1590 U/mg) (Additional file 7: Figure S5), the protein concentration of MAN330 expressed by T(-351)A plasmid was 6.4 g/L.

The expression levels of RuCelA and MAN330 were growth dependent (Fig. 5c, g), while they were less coupled with the growth in case of Est1E and Xyn-CDBFV (Fig. 5a, e). Although the expressions of different heterologous proteins are controlled by a same promoter and signal sequence, the association between protein production and growth can vary. Similar case has been seen in *S. cerevisiae* [42]. Differences at each step of protein production, such as synthesis, ER folding, and processing might lead to differences in growth-dependent production.

### Secretory expressions of lignocellulolytic enzymes in different carbon sources

**Fig. 6:**
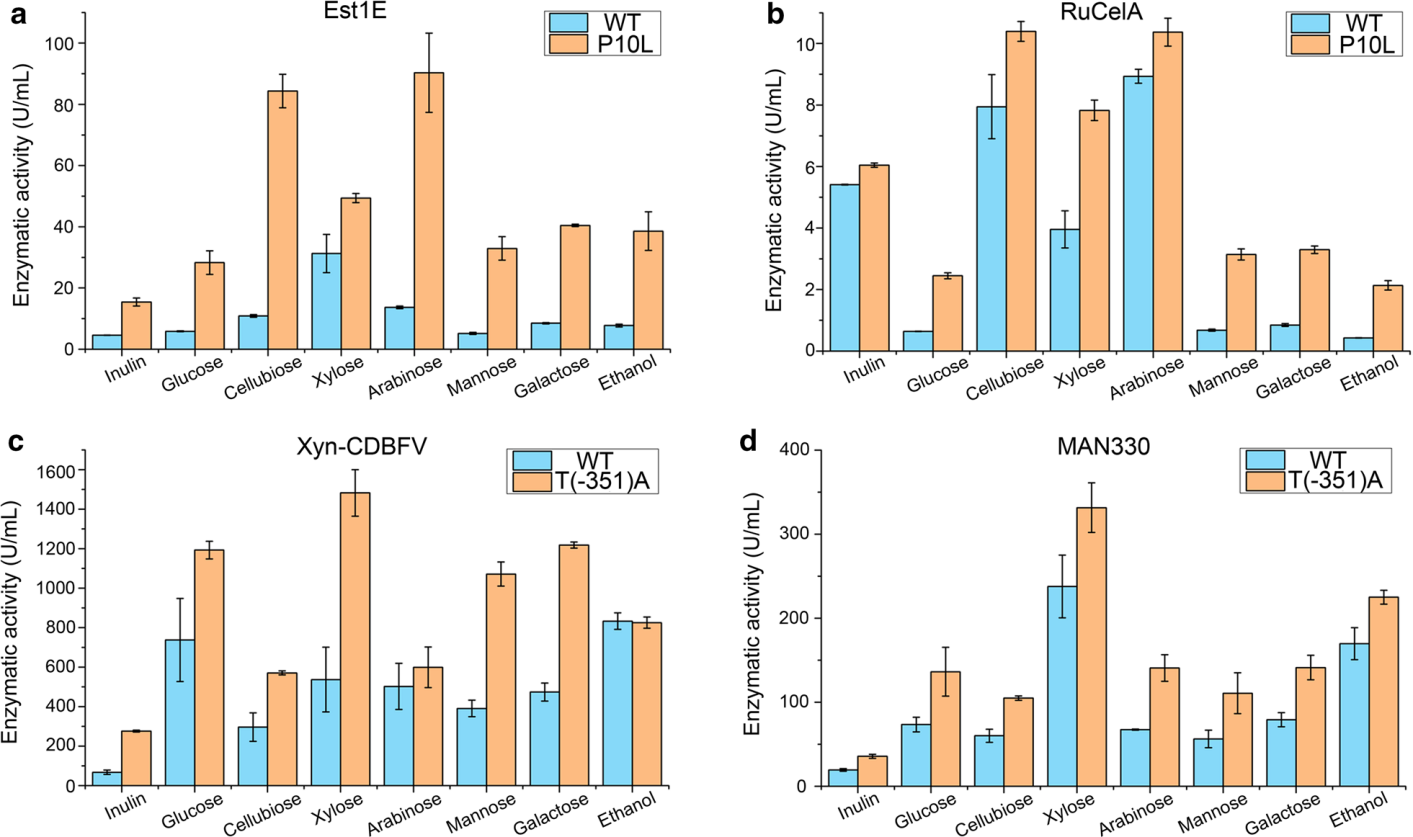
Expression of lignocellulolytic enzymes by WT or mutant plasmids on different carbon sources. Cells containing WT or mutant plasmid were grown in medium containing different carbon source for 72 h. Enzymatic activities in the supernatant of cells expressing Est1E (**a**), RuCelA (**b**), Xyn-CDBFV (**c**), or MAN330 (**d**) were measured. Values represent mean ± S.D. from three parallel cultures

The *INU1* promoter was induced by inulin and sucrose, but strongly repressed by glucose and lactose, and slightly inhibited by ethanol in *K. marxianus* CBS 6556 [18]. The *INU1* promoter used in this study originated from *K. marxianus* FIM-1 and showed 99% identity to that from *K. marxianus* CBS 6556. Saccharification of lignocellulose releases various types of sugars, including glucose, cellobiose, mannose, galactose, xylose, and l-arabinose. Therefore we investigated the effects of these sugars and ethanol. In general, activities of RuCelA, Est1E, Xyn-CDBFV and MAN330 produced by optimized promoter or signal sequence were higher than those by WT counterpart when using the above carbon sources (Fig. 6a–d).

Inulin was the poorest carbon source for expressing Est1E, Xyn-CDBFV and MAN330 (Fig. 6a, c, d), likely due to the relatively poor growth of cells in inulin medium in flasks (Additional file 8: Table S3). The expression of RuCelA in inulin was higher than that in glucose, mannose, galactose, and ethanol (Fig. 6b). l-arabinose and cellobiose were the best carbon sources in expression of RuCelA, since the activities expressed in both carbon sources reached 10 U/mL by means of optimized plasmids (Fig. 6b). Similarly, l-arabinose and cellobiose were the best carbon sources for expressing Est1E, which produced 90 U/mL and 84 U/mL of feruloyl esterase activities, respectively (Fig. 6a). Xylose was the best carbon source in expressing Xyn-CDBFV and MAN330 (Fig. 6c, d). The activities of Xyn-CDBFV and MAN330 produced by the optimized plasmid in xylose reached 1480 U/mL and 330 U/mL, respectively. Finally, ethanol, the end product of lignocellulosic fermentation, is also an efficient carbon source for our optimized plasmids. In case of Est1E and RuCelA, production in ethanol was comparable to that in glucose medium (Fig. 6a, b). In case of MAN330, a higher production was obtained by means of ethanol compared with glucose (Fig. 6d).

Compared to glucose, xylose is a more suitable carbon source for *K. marxianus* strains carrying our engineered plasmids to express lignocellulolytic enzymes. Pentose sugars constitute up to 40% of total sugars in lignocellulose hydrolysate [43]. Given the preference of utilizing glucose and slow consumption rate of xylose in *K. marxianus* [5, 44, 45], our optimized expression modules could be applied in engineering *K. marxianus* strains for constantly inducing high expression of lignocellulolytic enzymes during CBP.

## Discussion

Extension of the hydrophobic core of the *INU1* signal sequence or mutagenesis of *INU1* promoter increased secretory expression of endoglucanase RuCelA, endoxylanase Xyn-CDBFV, endomannanase MAN330, and feruloyl esterase Est1E by up to sixfold in *K. marxianus*. The peak activities of these lignocellulolytic enzymes expressed by engineered promoter and signal sequence in 5-L reactor were the highest activities reported for *K. marxianus* thus far.

Ethanol yield from lignocellulose requires large quantities of cellulases and hemicellulase to release fermentable sugars from plant feedstocks either by SHF or SSF [46]. Previous studies revealed *K. marxianus* simultaneously expressing a cocktail of cellulases-produced lower ethanol comparing to SSF due to the low yield of cellulolytic enzymes [16, 17, 47, 48]. Based on this consideration, our optimized promoter and signal peptide provide good candidates for elevating the expression of lignocellulolytic enzymes, cellulose hydrolysis, and ethanol yield in *K. marxianus*.

In industrial lignocellulolytic fungi, such as *T. reesei* and *Penicillium funiculosum*, only a small number of enzymes form the major part of lignocellulolytic enzymes arsenals. Cellobiohydrolase CEL7A accounts for around 60% of total cellulases secreted by *T. reesei* [49]. Cellobiohydrolase CBH1 and endoglucanase compose the major components of cellulases in *P. funiculosum* [50]. During the engineering of CBP strains, our optimized modules can be used to achieve high-level production of one or two preponderant lignocellulolytic enzymes, such as cellobiohydrolase or endoglucanase. Other less abundant cellulases can be expressed from different promoters, such as *CYC* or *TEF* [51], to compose a cellulase cocktail for cellulose hydrolysis and ethanol production.

It is notable that our recombinant *K. marxianus* strains were cultured in a highly aerobic condition in the fermentor. During the cellulosic ethanol production by *S. cerevisiae* CBP stains, recombinant strains are first cultured in an aerobic condition to obtain high cell density and to produce recombinant proteins. In the second stage, the precultured cells were transferred to fresh medium supplemented with cellulose substrate and cultured in an anaerobic condition to produce ethanol [52]. Thus, the same two-stage strategy might work with *K. marxianus* CBP strains harboring our optimized modules to produce ethanol from cellulose.

## Additional files

**Additional file 1: Table S1.** Plasmids used in this study.

**Additional file 2: Table S2.** Primers used in this study.

**Additional file 3: Figure S1.** Map of pUKDN132, a backbone vector for expressing heterologous proteins in *K. marxianus*. a) Schematic map of pUKDN132. b) MCS region of pUKDN132. Amino acids encoded by MCS region was labelled in red.

**Additional file 4: Figure S2.** Purification and specific activity of Est1E. Transformants containing pZP28 were grown in 5L fermentor for 56 h. 200 mL culture was centrifuged at 12, 000 rpm for 30 min at 4 degree. 100 mL supernatant was mixed with 400 mL 20mM Bis-Tris buffer (pH 6.6). 200 mL sample was purified by ion exchange chromatography with Q Bestarose FF column (AI0024, Bestchrom, Shanghai, China) and eluted with 20 mM Bis-Trish Buffer with 0~1 M NaCl. Fractions containing enzymatic activity were subjected to gel filtration (Superdex 200 Increase 10/300 GL, GE Healthcare, Illinois, USA) in an AKTA purifier 100 FPLC system (GE Healthcare). Flow rate was controlled at 0.5 mL/min in PBS buffer (137 mM NaCl, 2.7 mM KCl, 10mM Na_2_HPO_4_, 1,8 mM KH_2_PO_4_, pH 7.4). Peak of Est1E was eluted at 15 mL. The protein concentration of purified Est1E was 90 μg/mL, as measured by a BCA Protein Assay Kit (23250, Thermo, Illinois, USA). The enzymatic activity of purified Est1E was 215 U/mL and the specific activity of Est1E was 2400 U/mg. Supernatant of the culture, samples after purification by ion exchange and gel filtration were subjected to SDS-PAGE.

**Additional file 5: Figure S3.** Purification and specific activity of RuCelA. Transformants containing pZP52 were grown in 5L fermentor for 56 h. 200 mL culture was centrifuged at 12, 000 rpm for 30 min at 4 degree. The supernatant was purified by the ion exchange chromatography and gel filtration in the same procedure as described for Est1E. Peak of RuCelA was eluted at 16.3 mL. The protein concentration of purified RuCelA was 55 μg/mL, as measured by a BCA Protein Assay Kit. The enzymatic activity of purified RuCelA was 3.6 U/mL and the specific activity of RuCelA was 65 U/mg. Supernatant of the culture, samples after purification by ion exchange and gel filtration were subjected to SDS-PAGE.

**Additional file 6: Figure S4.** Deglycosylation of Xyn-CDBFV. Transformants containing pZP46 were grown in a 5L fermentor for 60 h. 1 mL culture was centrifuged at 12, 000 rpm for 5 min. Supernatant was collected and subjected to deglycosylation by Endo H (P0702S, NEB, USA) according to manufacturer’s manual. Sample before or after Endo H treatment was mixed with 5XSDS PAGE loading buffer and boiled. Samples were subjected to SDS-PAGE.

**Additional file 7: Figure S5.** Purification and specific activity of MAN330. Transformants containing pZP42 were grown in a 5L fermentor for 56 h. Culture was centrifuged at 12, 000 rpm for 30 min at 4 degree. 100 mL supernatant was mixed with 65 g (NH4)_2_SO4 and centrifuged at 12,000 rpm for 30 min at 4 degree. Supernatant was discarded. Precipitate was dissolved in 10 mL H_2_O and subjected to gel filtration (Superdex 75, 10/300, GL, GE Healthcare) in an AKTA purifier 100 FPLC system. Flow rate was controlled at 0.5 mL/min in PBS buffer (pH 7.4). Peak of MAN330 was eluted at 12 mL and fractions containing the peak were pooled. The protein concentration of purified MAN330 was 1mg/mL, as measured by a BCA Protein Assay Kit. The enzymatic activity of purified MAN330 was 1590 U/mL and the specific activity of MAN330 was 1590 U/mg. Precipitate dissolved in H_2_O and fractions eluted at 12 mL in gel filtration were subjected to SDS-PAGE.

**Additional file 8: Table S3.** OD_600_ of cells growing in different carbon sources.

## Abbreviations

CBP: consolidated bioprocessing
SHF: separate hydrolysis and fermentation
SSF: simultaneous saccharification and fermentation
WT: wild-type
MCS: multiple cloning sites
CNPF: 2-chloro-4-nitrophenyl ferulate
CNP: 2-chloro-4-nitrophenol
