# Supplementary material for: Improved secretory expression of lignocellulolytic enzymes in Kluyveromyces marxianus by promoter and signal sequence engineering
Source: Biotechnol Biofuels. 2018 Aug 29;11:235. doi: 10.1186/s13068-018-1232-7 (PMC6116501; doi:10.1186/s13068-018-1232-7)
Supplement: Supplementary file 1 — Additional file 1: Table S1. Plasmids used in this study. [file 13068_2018_1232_MOESM1_ESM.docx]

**Table S1**

Plasmids used in this study.

| Name^1^ | Description^2^ |
| --- | --- |
| pUKD | pKS, KD, URA3 |
| pZP12 | pMD18T-P_URA3_-*URA3* ORF-T_URA3_ |
| pZP13 | pMD18T- P_URA3_-T_URA3_ |
| pZP14 | pMD18T, P_INU1_, *INU1* ORF, T_INU1_ |
| pZP15 | pMD18T, P_INU1_, SS_INU1_, MCS, T_INU1_ |
| PUKDN132 | pKS, KD, P_INU1_, SS_INU1_, MCS, T_INU1_, URA3 |
| pZP17 | pKS, KD, P_INU1_, SS_INU1_, *Est1E*, T_INU1_, URA3 |
| pZP18 | pKS, KD, P_INU1_-M1, SS_INU1_, *Est1E*, T_INU1_, URA3 |
| pZP19 | pKS, KD, P_INU1_-M2, SS_INU1_, *Est1E*, T_INU1_, URA3 |
| pZP20 | pKS, KD, P_INU1_-M3, SS_INU1_, *Est1E*, T_INU1_, URA3 |
| pZP21 | pKS, KD, P_INU1_-SS_INU_(MM97), *Est1E*, T_INU1_, URA3 |
| pZP22 | pKS, KD, P_INU1_-UTRΔA, SS_INU1_, *Est1E*, T_INU1_, URA3 |
| pZP23 | pKS, KD, P_INU1_-A(-1104)T, SS_INU1_, *Est1E*, T_INU1_, URA3 |
| pZP24 | pKS, KD, P_INU1_-A(-566)T, SS_INU1_, *Est1E*, T_INU1_, URA3 |
| pZP25 | pKS, KD, P_INU1_-T(-351)A, SS_INU1_, *Est1E*, T_INU1_, URA3 |
| pZP26 | pKS, KD, P_INU1_-A(-266)G, SS_INU1_, *Est1E*, T_INU1_, URA3 |
| pZP27 | pKS, KD, P_INU1_-T(-233)A, SS_INU1_, *Est1E*, T_INU1_, URA3 |
| pZP28 | pKS, KD, P_INU1_, SS_INU1_-P10L, *Est1E*, T_INU1_, URA3 |
| pZP29 | pKS, KD, P_INU1_-T(-351)A-UTRΔA, SS_INU1_, *Est1E*, T_INU1_, URA3 |
| pZP30 | pKS, KD, P_INU1_-T(-351)A, SS_INU1_-P10L, *Est1E*, T_INU1_, URA3 |
| pZP31 | pKS, KD, P_INU1_-T(-351)A-UTRΔA, SS_INU1_-P10L, *Est1E*, T_INU1_, URA3 |
| pZP32 | pKS, KD, P_INU1_, SS_INU1_, *Est1E*-*His_6_*, T_INU1_, URA3 |
| pZP33 | pKS, KD, P_INU1_, SS_INU1_-P10L, *Est1E*-*His_6_*, T_INU1_, URA3 |
| pZP34 | pKS, KD, P_INU1_, SS_INU1_-P10I, *Est1E*, T_INU1_, URA3 |
| pZP35 | pKS, KD, P_INU1_, SS_INU1_-P10G, *Est1E*, T_INU1_, URA3 |
| pZP36 | pKS, KD, P_INU1_, SS_INU1_-P10S, *Est1E*, T_INU1_, URA3 |
| pZP37 | pKS, KD, P_INU1_, SS_INU1_-P10K, *Est1E*, T_INU1_, URA3 |
| pZP38 | pKS, KD, P_INU1_, SS_INU1_-P10D, *Est1E*, T_INU1_, URA3 |
| pZP39 | pKS, KD, P_INU1_, SS_KL-INU1_, *Est1E*, T_INU1_, URA3 |
| pZP40 | pKS, KD, P_INU1_, SS_KL-INU1_-P10L, *Est1E*, T_INU1_, URA3 |
| pZP41 | pKS, KD, P_INU1_, SS_INU1_, *MAN330*, T_INU1_, URA3 |
| pZP42 | pKS, KD, P_INU1_-T(-351)A, SS_INU1_, *MAN330*, T_INU1_, URA3 |
| pZP43 | pKS, KD, P_INU1_-UTRΔA, SS_INU1_, *MAN330*, T_INU1_, URA3 |
| pZP44 | pKS, KD, P_INU1_, SS_INU1_-P10L, *MAN330*, T_INU1_, URA3 |
| pZP45 | pKS, KD, P_INU1_, SS_INU1_, *Xyn-CDBFV*, T_INU1_, URA3 |
| pZP46 | pKS, KD, P_INU1_-T(-351)A, SS_INU1_, *Xyn-CDBFV*, T_INU1_, URA3 |
| pZP47 | pKS, KD, P_INU1_-UTRΔA, SS_INU1_, *Xyn-CDBFV*, T_INU1_, URA3 |
| pZP48 | pKS, KD, P_INU1_, SS_INU_-P10L, *Xyn-CDBFV*, T_INU1_, URA3 |
| pZP49 | pKS, KD, P_INU1_, SS_INU1_, *RuCel1A*, T_INU1_, URA3 |
| pZP50 | pKS, KD, P_INU1_-T(-351)A, SS_INU1_, *RuCel1A*, T_INU1_, URA3 |
| pZP51 | pKS, KD, P_INU1_-UTRΔA, SS_INU1_, *RuCel1A*, T_INU1_, URA3 |
| pZP52 | pKS, KD, P_INU1_, SS_INU1_-P10L, *RuCel1A*, T_INU1_, URA3 |

^1^ All plasmids except pUKD were constructed in this study.

^2^ Meaning of abbreviations: pKS, pBluescript II KS(+); P_INU1_, inulinase promoter; T_INU1_, inulinase terminator; SS_INU1_, inulinase signal sequence; MCS, multiple cloning sites; SS_KL-INU1_, inulinase signal sequence from *Kluyveromyces lactis*.
