# Supplementary material for: Improved secretory expression of lignocellulolytic enzymes in Kluyveromyces marxianus by promoter and signal sequence engineering
Source: Biotechnol Biofuels. 2018 Aug 29;11:235. doi: 10.1186/s13068-018-1232-7 (PMC6116501; doi:10.1186/s13068-018-1232-7)
Supplement: Supplementary file 2 — Additional file 2: Table S2. Primers used in this study. [file 13068_2018_1232_MOESM2_ESM.docx]

**Table S2**

Primers used in this study.

| Name | Sequence^1^ |
| --- | --- |
| URA3-F | ATAATGCGTA TAGTCAAATC TGGCC |
| URA3-R | GAAGTCCTTC ATTTGCTTTT GTTCC |
| URA3Δ-F^2^ | CATCAGCTAGCCCCATAAACTAATCAAGGTTCGAGTGTACTCGGATCAGAAG |
| URA3Δ-R^3^ | CTTCTGATCCGAGTACACTCGAACCTTGATTAGTTTATGGGG CTAGCTGATG |
| URA3-R2 | CATCTTTCGA CCAAGTTGGA GTATAG |
| INU1-F | TGCCGATTCGCACGCTGCAACCGCGGCACAAACACAAACACAAACACAAAAACGC |
| INU1-R | AATCAGAATTCGACCGATCCTAGAATGTTGGTCAGATGTGATGTACAC |
| MCS-F | TCAGTGATCAATTACAAGAGAGACGGTGACCCCGGGACTAGTGCGGCCGCTTAAGGCCGCAAGCTTTGATCTGATCTGCTTACTTT ACTAACGACA |
| MCS-R | TGTCGTTAGTAAAGTAAGCAGATCAGATCAAAGCTTGCGGCCTTAAGCGGCCGCACTAGTCCCGGGGTCACCGTCTCTCTTGTAAT TGATCACTGA |
| PUKD-F | GGATCGGTCG AATTCTGATTGGAAAGACC |
| PUKD-R | TTGCAGCGTG CGAATCGGCACGGTAATGATTCG |
| Est1E-F | ACGGTGACCCCGGGACTAGTTATATTGATTGTGACGGTATAAAATTAAATG |
| Est1E-R | TTGCGGCCTTAAGCGGCCGCTTATTTAGCAATCTGCTCAAGCATAAATTC |
| His6-R | TTGCGGCCTTAAGCGGCCGCTTAATGGTGGTGATGATGGTG TTTAGCAATCTGCTCAAGCATAAA |
| M1-F | TTCGCACGCTGCAACCGCGGAAATACACAGACGAGCGCGCACAG |
| M2-F | TTCGCACGCTGCAACCGCGGGAATTCTCAAACCGAAATGGGGCG |
| M3-F | TTCGCACGCTGCAACCGCGGGGAAAAACTTTTCCTGTGTTAATCC |
| MP-R | CACAATCAATATAACTAGTCCCGGG |
| MutP-F | ATTCGCACGCTGCAACCGCGG |
| MutR-R | ATAACTAGTCCCGGGGTCACCGTCT |
| UTRΔA-F | GAATTACTACTGTGTGTAACGGTTATATTTCGGTTAGATATGAAGTTAGCATACTCCCTCTTG |
| UTRΔA-R | CAAGAGGGAGTATGCTAACTTCATATCTAACCGAAATATAACC GTTACACACA GTAGTAATTC |
| A(-1104)TF | ACACAAAAACGCTATATTATGCACACAAGG |
| A(-1104)TR | CCTTGTGTGCATAATATAGCGTTTTTGTGT |
| A(-566)T-F | GAATCACACACACACTCACAGTTTATTGGA G |
| A(-566)T-R | CTCCAATAAACTGTGAGTGTGTGTGTGATT C |
| T(-351)A-F | AAACTTTTCCTGTGTAAATCCGGCCGTGCG |
| T(-351)A-R | CGCACGGCCGGATTTACACAGGAAAAGTTT |
| A(-266)G-F | AATTCACATATAAAAAGCGTGTCTCGAGATCTCAAAGTCT |
| A(-266)G-R | AGACTTTGAGATCTCGAGACACGCTTTTTATATGTGAATT |
| T(-233)A-F | CTCCCTTGAATCGTGATTGCCAGTTGTAAC |
| T(-233)A-R | GTTACAACTGGCAATCACGATTCAAGGGAG |
| P10L-F | AGCATACTCCCTCTTGCTTCTATTGGCAGGAGTCAGTGCT |
| P10L-R | AGCACTGACTCCTGCCAATAGAAGCAAGAGGGAGTATGCT |
| P10I-F | AGCATACTCCCTCTTGCTTATCTTGGCAGGAGTCAGTGCT |
| P10I-R | AGCACTGACTCCTGCCAAGATAAGCAAGAGGGAGTATGCT |
| P10G-F | AGCATACTCCCTCTTGCTTGGTTTGGCAGGAGTCAGTGCT |
| P10G-R | AGCACTGACTCCTGCCAAACCAAGCAAGAGGGAGTATGCT |
| P10S-F | AGCATACTCCCTCTTGCTTTCCTTGGCAGG AGTCAGTGCT |
| P10S-R | AGCACTGACTCCTGCCAAGGAAAGCAAGAGGGAGTATGCT |
| P10K-F | AGCATACTCC CTCTTGCTTAAGTTGGCAGGAGTCAGTGCT |
| P10K-R | AGCACTGACTCCTGCCAACTTAAGCAAGAG GGAGTATGCT |
| P10D-F | AGCATACTCCCTCTTGCTTGATTTGGCAGG AGTCAGTGCT |
| P10D-R | AGCACTGACTCCTGCCAAATCAAGCAAGAG GGAGTATGCT |
| KLSS^4^ | CTCGAGATCTCAAAGTCTCCCTTGAATCGTGTTTGCCAGT GTGACACATTTAATTTTTTTTTTGTTAGATATGTTAAAGTTGTTGTCATTAATGGTGCCTTTGGCATCTGCTGCTGTCATCCACCGCCGTGATGCTCCCGGGACTAGTTATATTGATTGTGAC |
| KL-P10L-F | TGTTAAAGTTGTTGTCATTAATGGTGTTGTTGGCATCTGCTGCTGTCATCCAC |
| KL-P10L-R | GTGGATGACAGCAGCAGATGCCAACAACACCATTAATGACAACAACTTTAACA |
| Man-F | ACGGTGACCCCGGGACTAGTGTTGATGGCAATACGTTATATGACG |
| Man-R | TTGCGGCCTTAAGCGGCCGCCTATGTAAATACGGTGGATGGTTTGGAGG |
| Xyn-F | ACGGTGACCCCGGGACTAGTTCTTCTGGTTTCTACGTTGATGGCAATACG |
| Xyn-R | TTGCGGCCTTAAGCGGCCGCTCAATCACCAATGTAAAC |
| CelA-F | ACGGTGACCCCGGGACTAGTTGTATGAAGAAAACTCTACTTTTTGTTTG |
| CelA-R | TTGCGGCCTTAAGCGGCCGCTTATTTGGCAATGTGCGTTTTACCG |
| Est1-RT-F^5^ | AGAGCGACGGCAAGTTTGAGGA |
| Est1-RT-R^5^ | CAGCAGGTGATAGTGGAATGAGTG |
| LEU2-F^5^ | GGGCTTACTACCATCCGCTTCT |
| LEU2-R^5^ | TAACGGCTTCTTCAACGGCTCT |
| 18S-F^5^ | TAGTTGGTGGAGTGATTTGTCTGC |
| 18S-R^5^ | CTCGCTGGCTCCGTCAGTGTAG |

1. Sites mutated by PCR mutagenesis are underlined.
2. Sequence in red is same as -33~-7 bp upstream of *URA3* ORF. Sequence in green is same as 23~47bp downstream of *URA3* ORF.
3. Reverse primer is complementary to the forward primer.
4. KLSS was liner double strand DNA synthesized by Genewiz. KLSS was used as a megaprimer to replace inulinase signal sequence of *K. marxianus* in pZP17 by that of *K. lactis* in mutagenesis PCR. Sequence encoding inulinase signal sequence from *K. lactis* is in red, which was extracted from the genome of NRRL Y-1140 on NCBI. KLSS stands for inulinase signal sequence from *K. lactis*.
5. Primers used in real-time PCR.
