# Supplementary figures and images for: Improved secretory expression of lignocellulolytic enzymes in Kluyveromyces marxianus by promoter and signal sequence engineering
Source: Biotechnol Biofuels. 2018 Aug 29;11:235. doi: 10.1186/s13068-018-1232-7 (PMC6116501; doi:10.1186/s13068-018-1232-7)

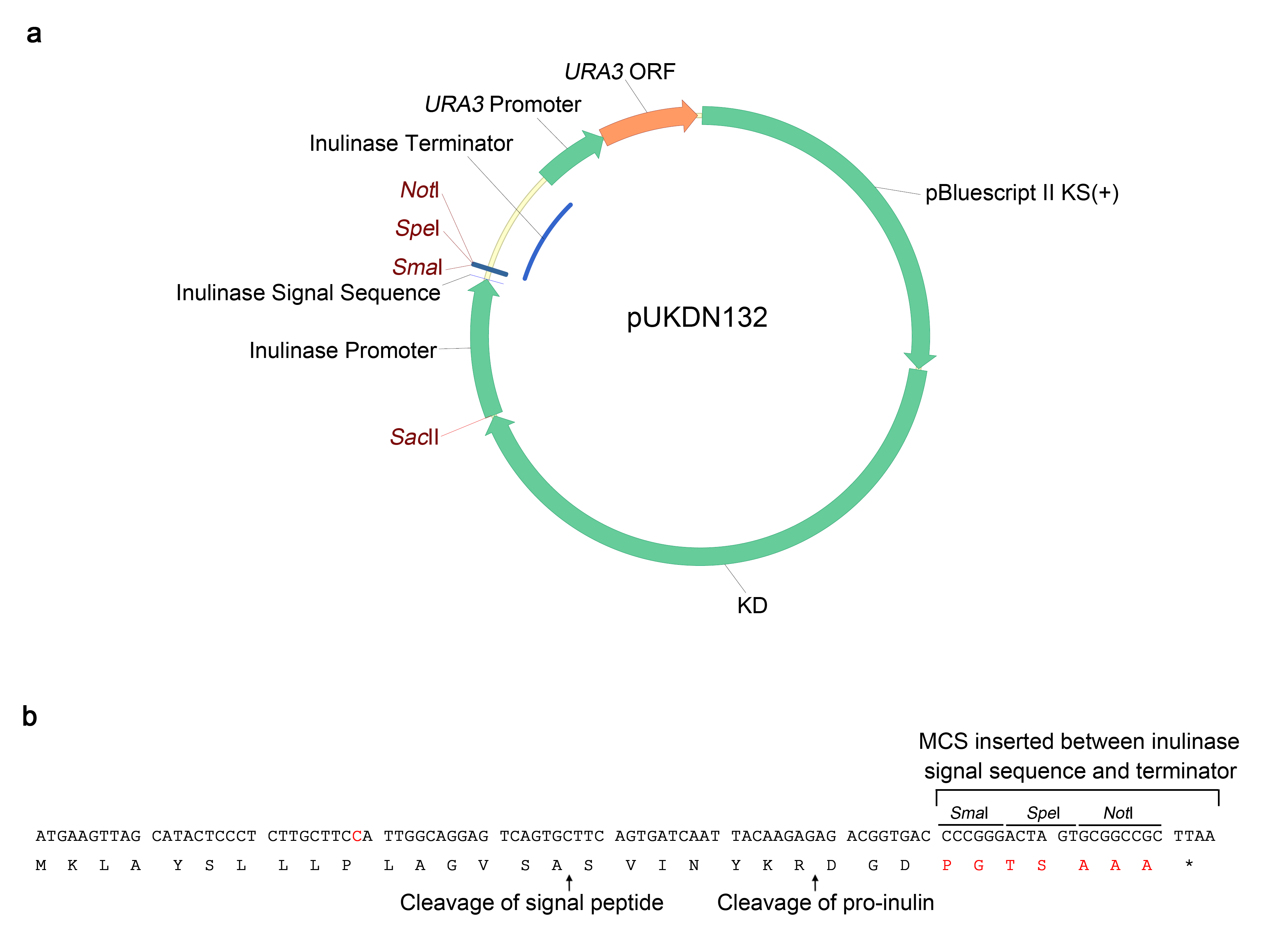

Supplement: Supplementary file 3 — Additional file 3: Figure S1. Map of pUKDN132, a backbone vector for expressing heterologous proteins in K. marxianus. a) Schematic map of pUKDN132. b) MCS region of pUKDN132. Amino acids encoded by MCS region was labelled in red. [file 13068_2018_1232_MOESM3_ESM.tif]

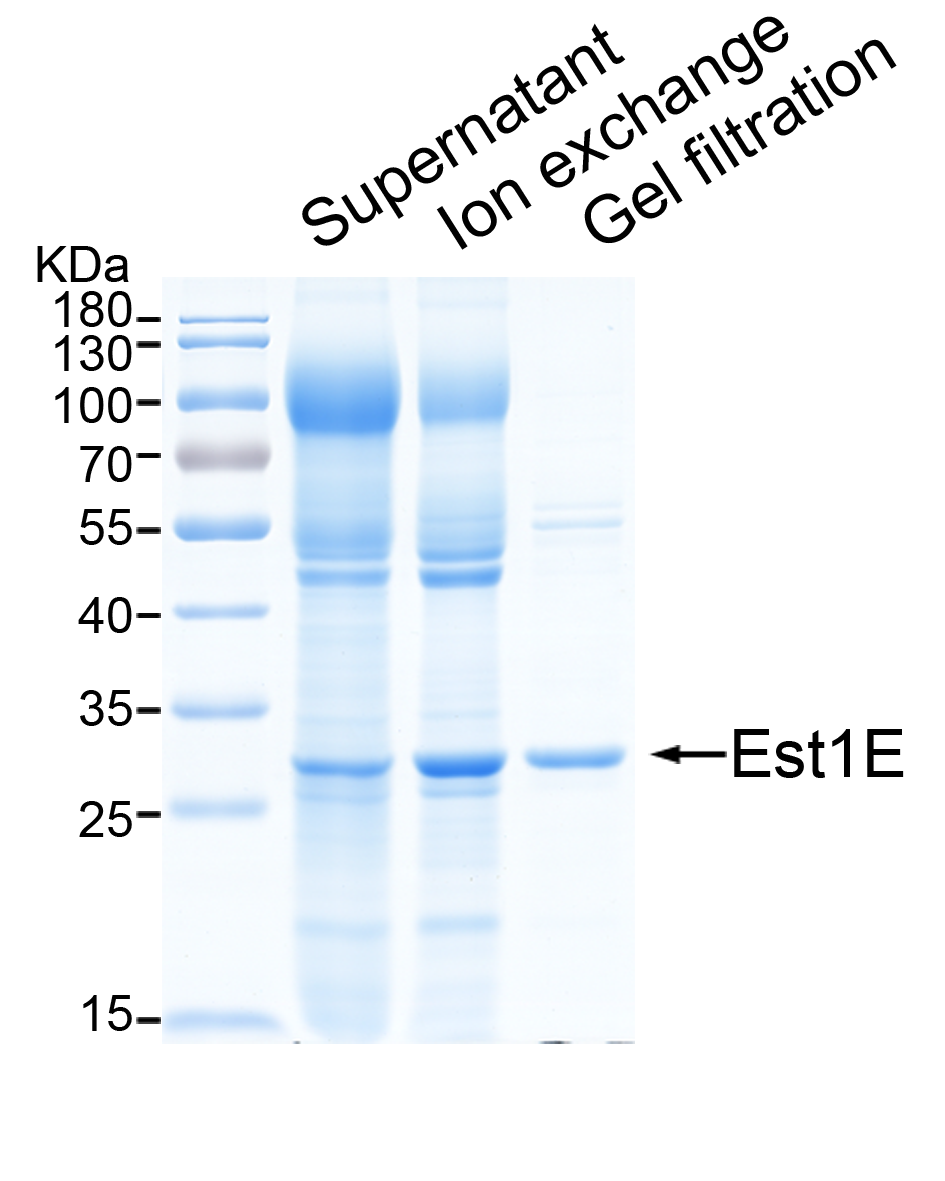

Supplement: Supplementary file 4 — Additional file 4: Figure S2. Purification and specific activity of Est1E. Transformants containing pZP28 were grown in 5L fermentor for 56 h. 200 mL culture was centrifuged at 12, 000 rpm for 30 min at 4 degree. 100 mL supernatant was mixed with 400 mL 20mM Bis-Tris buffer (pH 6.6). 200 mL sample was purified by ion exchange chromatography with Q Bestarose FF column (AI0024, Bestchrom, Shanghai, China) and eluted with 20 mM Bis-Trish Buffer with 0~1 M NaCl. Fractions containing enzymatic activity were subjected to gel filtration (Superdex 200 Increase 10/300 GL, GE Healthcare, Illinois, USA) in an AKTA purifier 100 FPLC system (GE Healthcare). Flow rate was controlled at 0.5 mL/min in PBS buffer (137 mM NaCl, 2.7 mM KCl, 10mM Na2HPO4, 1,8 mM KH2PO4, pH 7.4). Peak of Est1E was eluted at 15 mL. The protein concentration of purified Est1E was 90 μg/mL, as measured by a BCA Protein Assay Kit (23250, Thermo, Illinois, USA). The enzymatic activity of purified Est1E was 215 U/mL and the specific activity of Est1E was 2400 U/mg. Supernatant of the culture, samples after purification by ion exchange and gel filtration were subjected to SDS-PAGE. [file 13068_2018_1232_MOESM4_ESM.tif]

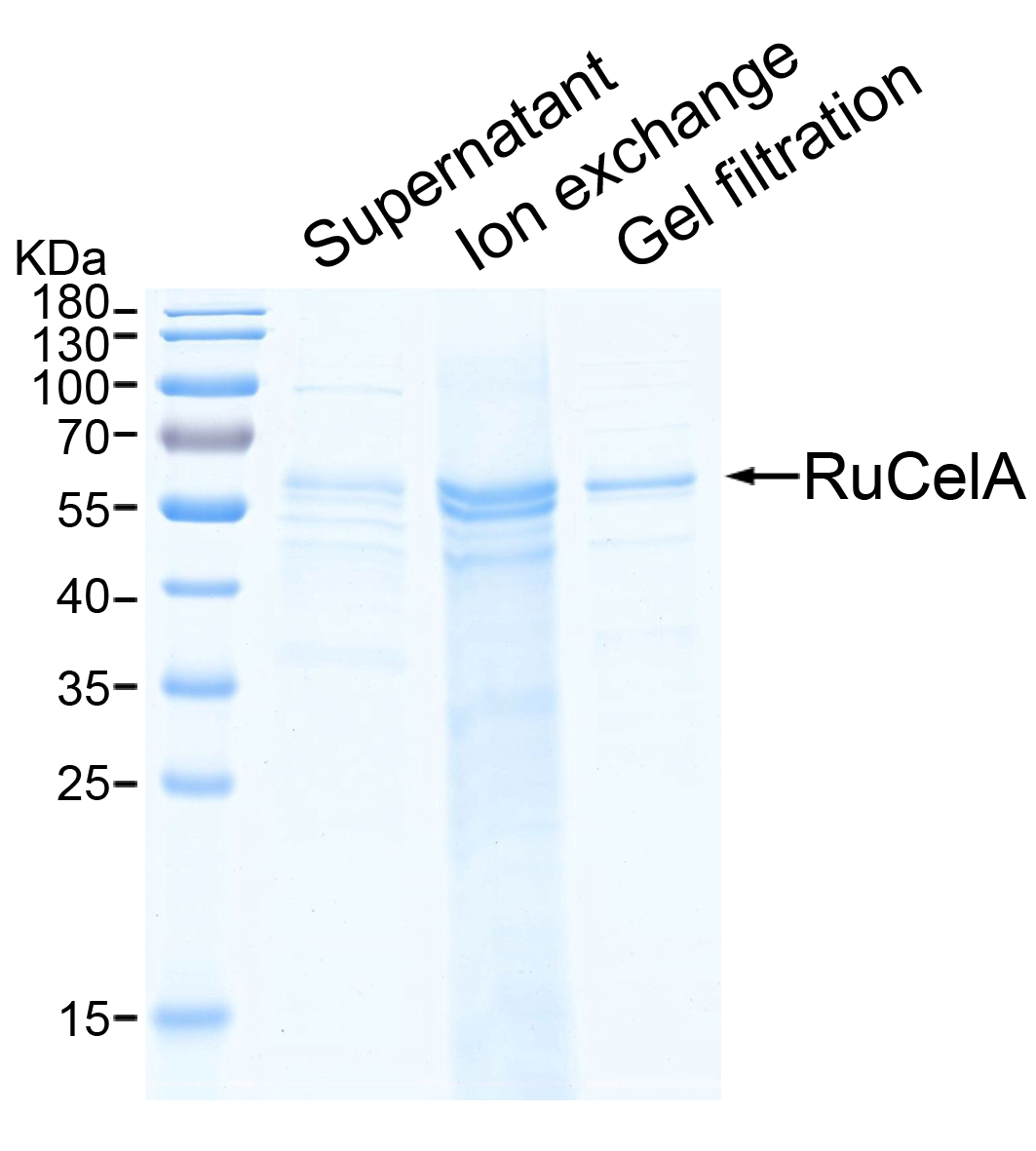

Supplement: Supplementary file 5 — Additional file 5: Figure S3. Purification and specific activity of RuCelA. Transformants containing pZP52 were grown in 5L fermentor for 56 h. 200 mL culture was centrifuged at 12, 000 rpm for 30 min at 4 degree. The supernatant was purified by the ion exchange chromatography and gel filtration in the same procedure as described for Est1E. Peak of RuCelA was eluted at 16.3 mL. The protein concentration of purified RuCelA was 55 μg/mL, as measured by a BCA Protein Assay Kit. The enzymatic activity of purified RuCelA was 3.6 U/mL and the specific activity of RuCelA was 65 U/mg. Supernatant of the culture, samples after purification by ion exchange and gel filtration were subjected to SDS-PAGE. [file 13068_2018_1232_MOESM5_ESM.tif]

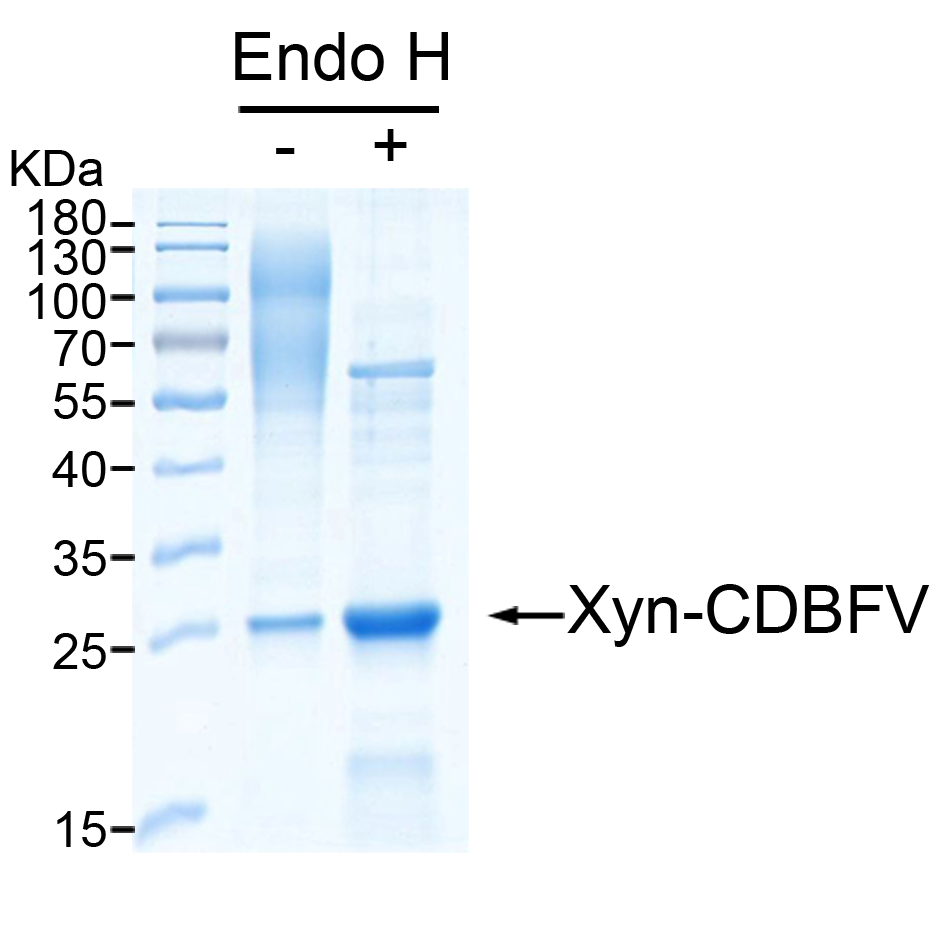

Supplement: Supplementary file 6 — Additional file 6: Figure S4. Deglycosylation of Xyn-CDBFV. Transformants containing pZP46 were grown in a 5L fermentor for 60 h. 1 mL culture was centrifuged at 12, 000 rpm for 5 min. Supernatant was collected and subjected to deglycosylation by Endo H (P0702S, NEB, USA) according to manufacturer’s manual. Sample before or after Endo H treatment was mixed with 5XSDS PAGE loading buffer and boiled. Samples were subjected to SDS-PAGE. [file 13068_2018_1232_MOESM6_ESM.tif]

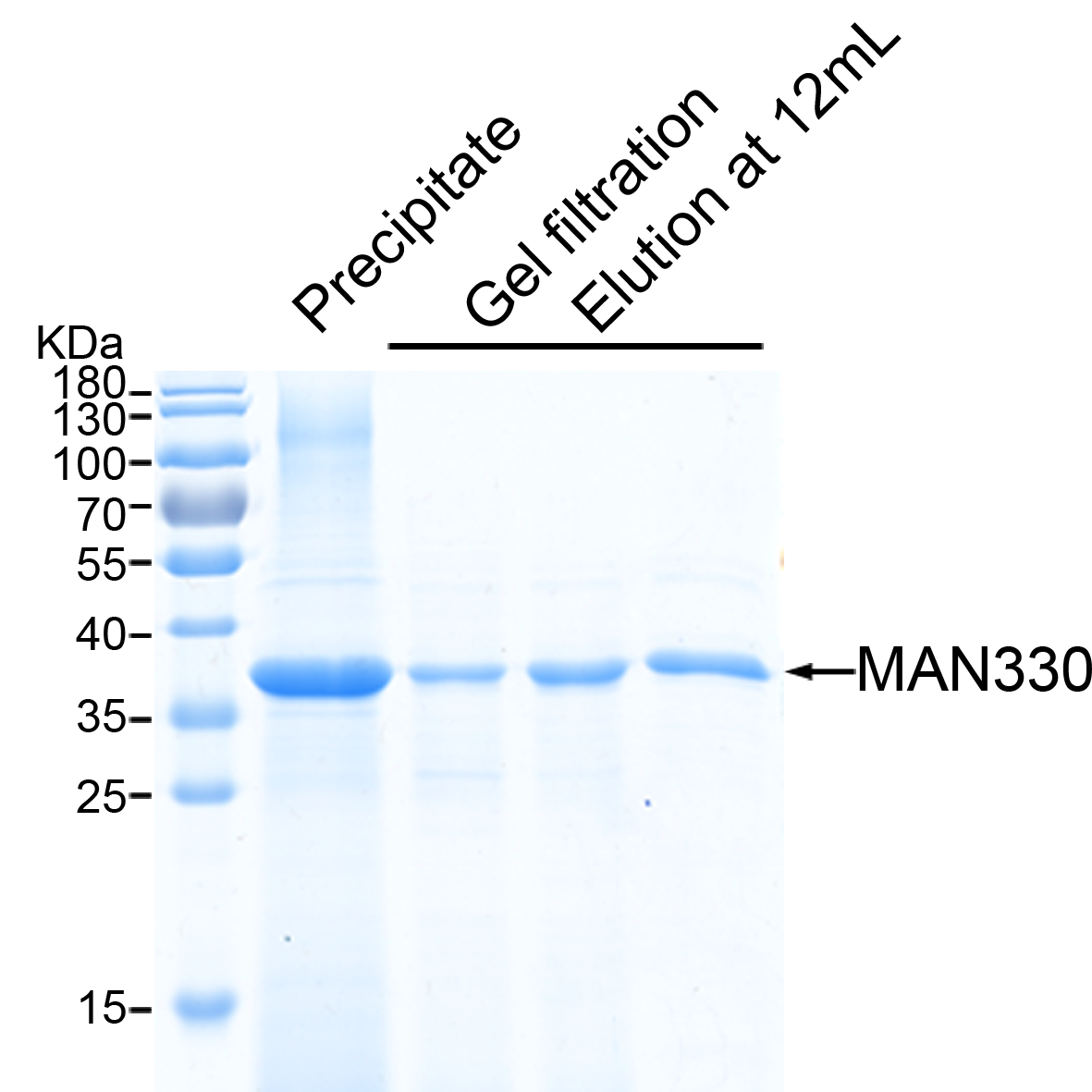

Supplement: Supplementary file 7 — Additional file 7: Figure S5. Purification and specific activity of MAN330. Transformants containing pZP42 were grown in a 5L fermentor for 56 h. Culture was centrifuged at 12, 000 rpm for 30 min at 4 degree. 100 mL supernatant was mixed with 65 g (NH4)2SO4 and centrifuged at 12,000 rpm for 30 min at 4 degree. Supernatant was discarded. Precipitate was dissolved in 10 mL H2O and subjected to gel filtration (Superdex 75, 10/300, GL, GE Healthcare) in an AKTA purifier 100 FPLC system. Flow rate was controlled at 0.5 mL/min in PBS buffer (pH 7.4). Peak of MAN330 was eluted at 12 mL and fractions containing the peak were pooled. The protein concentration of purified MAN330 was 1mg/mL, as measured by a BCA Protein Assay Kit. The enzymatic activity of purified MAN330 was 1590 U/mL and the specific activity of MAN330 was 1590 U/mg. Precipitate dissolved in H2O and fractions eluted at 12 mL in gel filtration were subjected to SDS-PAGE. [file 13068_2018_1232_MOESM7_ESM.tif]
