# Supplementary material for: Improved secretory expression of lignocellulolytic enzymes in Kluyveromyces marxianus by promoter and signal sequence engineering
Source: Biotechnol Biofuels. 2018 Aug 29;11:235. doi: 10.1186/s13068-018-1232-7 (PMC6116501; doi:10.1186/s13068-018-1232-7)
Supplement: Supplementary file 8 — Additional file 8: Table S3. OD600 of cells growing in different carbon sources. [file 13068_2018_1232_MOESM8_ESM.docx]

**Table S3**

**OD_600_ of cells growing in different carbon sources**

| Enzyme | plasmid | Carbon source | | | | | | | |
| --- | --- | --- | --- | --- | --- | --- | --- | --- | --- |
|  |  | Inulin | Glucose | Cellubiose | Xylose | Arabinose | Mannose | Galactose | Ethanol |
| Est1E | WT | 13.1 | 30 | 14 | 29 | 19 | 24.9 | 31.4 | 23 |
|  | P10L | 10.2 | 29.7 | 15.5 | 30 | 22.1 | 27.5 | 33 | 20.2 |
| RuCelA | WT | 11.9 | 31 | 18.8 | 26.7 | 21.6 | 25.5 | 28.5 | 23.5 |
|  | P10L | 11.8 | 29.8 | 19 | 28.7 | 20.9 | 27.4 | 26.3 | 24.6 |
| Xyn-CDBFV | WT | 15.8 | 23.8 | 16.7 | 21.9 | 20.1 | 21.6 | 21.8 | 20 |
|  | T(-351)A | 15.6 | 23 | 16.5 | 22.9 | 19.2 | 21.3 | 21 | 19.5 |
| MAN330 | WT | 14 | 25.5 | 27.5 | 27.2 | 22.4 | 25.5 | 26 | 23.8 |
|  | T(-351)A | 13.8 | 27.8 | 20.6 | 26.4 | 22.4 | 25.3 | 26.6 | 22.4 |

Cells containing WT or mutant plasmid were grown in medium containing different carbon source for 72h. Average OD_600_ of three parallel culture was listed here.
